# A Critical Review and Scientific Prospective on Contraceptive Therapeutics from Ayurveda and Allied Ancient Knowledge

**DOI:** 10.3389/fphar.2021.629591

**Published:** 2021-06-03

**Authors:** Narendra Bhatt, Manasi Deshpande

**Affiliations:** ^1^ CRIA Consultant Pvt. Ltd. Mumbai, Mumbai, India; ^2^ Department of DravyagunaVigynan, Bharati Vidyapeeth Deemed to be University, College of Ayurved, Pune, India

**Keywords:** natural contraceptive, herbal contraceptive, ayurved contraceptive, reproductive health and traditional medicine, contraceptive traditions

## Abstract

Commonly used synthetic or prescribed hormonal drugs are known to interfere with the endocrine system and may have adverse reproductive, neurological, developmental, and metabolic effects in the body. These may also produce adverse effects such as polycystic ovarian disorder, endometriosis, early puberty, infertility or toxicity to gonads, testicular germ cell cancer, breast or prostate cancer, brain developmental problems, and even birth defects. Globally, the emergence of renewed interest in natural products for reproductive health is on the rise, which offers opportunities for new contraceptive developments. The search for alternate, safer contraceptive products or agents of natural origin is of scientific interest. Ayurvedic classical texts offer knowledge and information about the reproductive function and therapeutics including those for enhancement and limiting male and female fertility. Review of ancient, medieval, and recent—including texts on erotica that provide information on approaches and large numbers of formulations and drugs of plant, mineral or animal origin—claimed to have sterilizing, contraceptive, abortifacient, and related properties is presented. Few among these are known to be toxic and few are not so common. However, most of the formulations, ingredients, or modes of administration have remained unattended to, due to issues related to consumer compliance and limitations of standardization and lack of appropriate validation modalities. Several of these ingredients have been studied for their phytoconstituents and for the variety of pharmacological activities. Efforts to standardize several classical dosage forms and attempts to adapt to modern technologies have been made. List of formulations, ingredients, and their properties linked with known constituents, pharmacological, biological, and toxicity studies have been provided in a series of tables. The possible effectiveness and safety of selected formulations and ingredients have been examined. Suggestions based on new drug delivery systems integrated with advances in biotechnology, to provide prospects for new therapeutics for contraception, have been considered. Ayurveda is built on a holistic paradigm of biological entity rather than limited gonadal functions. Graphic presentation of a few carefully chosen possibilities has been depicted. New approaches to standardization and ethnopharmacological validation of natural contraceptive therapeutics may offer novel mechanisms and modalities and therapeutic opportunities to satisfy unmet needs of contraception.

## Introduction

The world population is expected to reach more than 11 billion by 2050 (Census of India, 2011). Population in the world is currently (2020) growing at a rate of around 1.05% per year. The current average population increase is estimated at 81 million people per year and current world population is 7.9 billion as of March 2021 (World Population Clock, 2021). This burgeoning population particularly in developing countries is a matter of concern for social, economic, and environmental reasons in terms of providing food, shelter, and life. The challenge of dealing with an ever-increasing population has been dealt with largely by conventional medicine using different methods of contraception such as oral contraceptive pills, intrauterine contraceptive devices, and barrier devices. These devices, techniques, and drugs seem to have been efficiently practiced for contraception but with many reported adverse effects as well as failure resulting in unwanted pregnancy. (Dutta, 2013).

## Birth Control History

Technically, birth control can be defined as the methods, procedures, or practices that are implemented to prevent conception leading to pregnancy in women. The term can be associated with contraception and family planning where knowledge about birth control is equally important.

The Egyptian Ebers Papyrus from 1550 BCE and the Kahun Papyrus from 1850 BCE have some of the earliest documented descriptions of birth control: the use of honey, acacia leaves, and lint to be placed in the vagina to block sperm. (Lipsey et al., 2005; Cuomo, 2010).

In medieval Europe, any effort to halt pregnancy was deemed immoral by the Catholic Church, (Cuomo, 2010), although it is believed that women of the time still used a number of birth control measures such as coitus interrupts and inserting lily root and rue into the vagina. Women in the middle ages were also persuaded to tie weasel—a small wild animal—testicles around their thighs during sex to prevent pregnancy. The oldest condoms discovered to date were recovered in the ruins of Dudley Castle in England and date back to 1640. They were made of animal gut and were most likely used to prevent the spread of sexually transmitted diseases during the English Civil War (Jon, 2012). Casanova, living in 18th-century Italy, described the use of a lambskin covering to prevent pregnancy; however, condoms only became widely available in the 20th century (Cuomo, 2010).

### Modern Methods to Control Fertility (World Health Organization, 2020)

**FIGURE 1:**
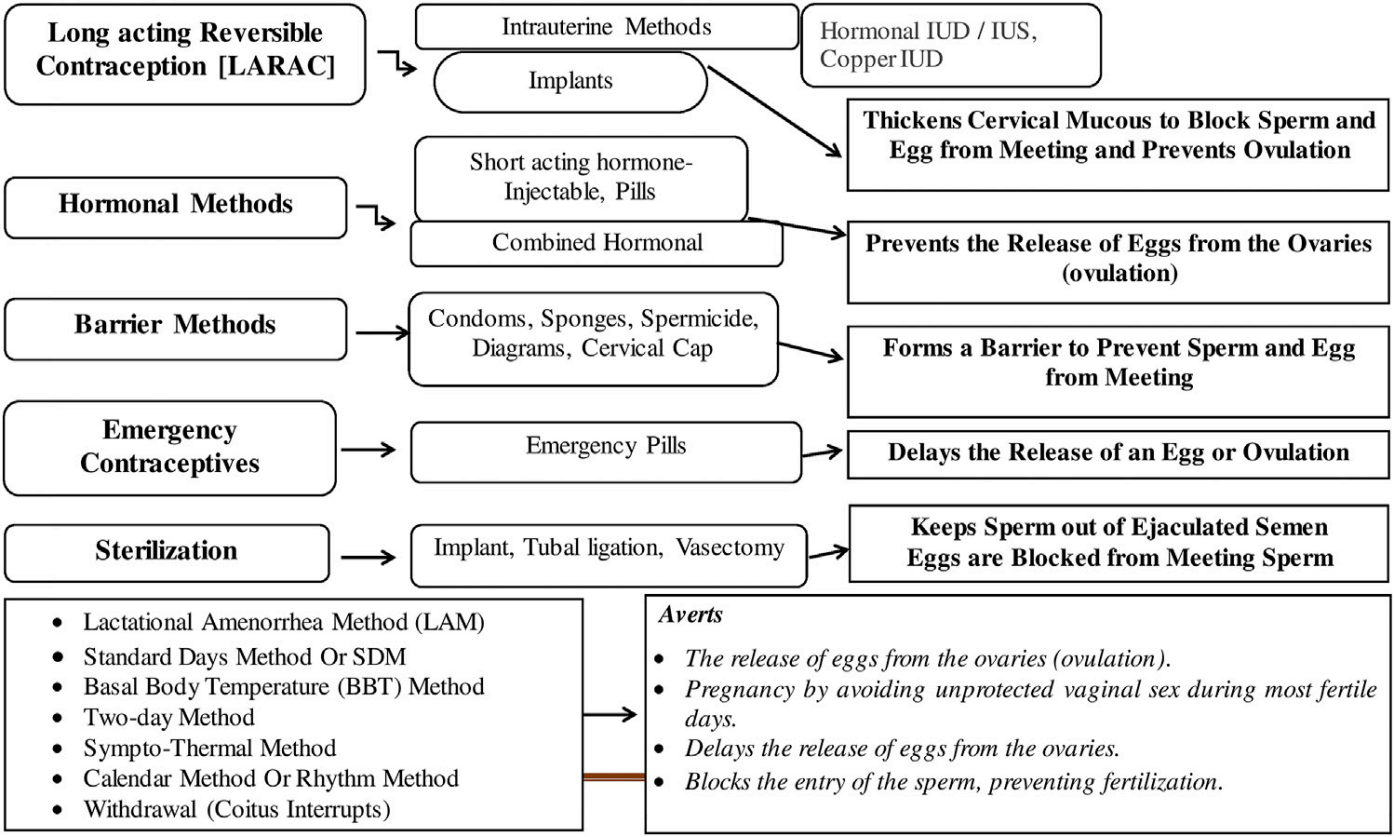
Modern methods of birth control.

Several methods currently used to curb for contraception are presented (Figure 1).

## Adverse Effects

Commonly used synthetic or prescribed hormonal drugs are known to interfere with the endocrine system and may have adverse reproductive, neurological, developmental, and metabolic effects in the body. These may cause polycystic ovarian disorder, endometriosis, early puberty, infertility, toxicity to gonads, testicular germ cell cancer, breast or prostate cancer, brain developmental problems, and even birth defects. The search for alternate and safer means/drugs to prevent birth is an open-ended area of scientific research. It is always an appealing idea to further research to develop contraceptive drugs of natural origin that have high efficacy without any adverse effects on the reproductive system.

## Unmet Needs

According to a recent report from the Guttmacher Institute, 214 million women of reproductive age in the developing world who want to avoid pregnancy are not using a modern contraceptive method. These women are considered to have an “unmet need” for modern contraception, with 59 million relying on traditional methods such as abstinence and withdrawal and 155 million simply using no contraception at all. (Elizabeth et al., 2020).

India’s total fertility rate (TFR) may have declined significantly over the years, but there remain significant challenges in family planning according to new research. In an Economic and Political Weekly article, Purushottam M. Kulkarni of Jawaharlal Nehru University suggested that there is a significant unmet need for contraception in India. Data from National Family Health Surveys (NFHS) have shown that while there was a decline in the unmet need for contraceptive services from 1992-93 (NFHS-1) through to 2005-06 (NFHS-3), and between 2005-06 and 2015-16 (NFHS-4), there has not been any significant improvement in access to contraception. (Mint, 2020).

## Significance of Review

Despite obvious success, the rise in population continues to remain a medical challenge due to reasons of social, economic, personal, and biological consequences. Though well-established contraceptive drugs and measures have been utilized, the long term and excessive use of hormonal contraceptives are of serious concerns due to their probable adverse effects. There is need to explore the alternative or new possibilities.

The search for an effective and safe contraceptive agent remains a challenge. Contraceptive drugs of natural origin are of all-time research interests. Traditional systems of medicine like Ayurveda address all issues related to health and illnesses based on the principle of equilibrium between the biosphere and cosmosphere, which include reproductive phenomenon. Ayurvedic pharmacopoeia has formulations and ingredients that are attributed to affect coitus, spermatogenesis, and ovulation, uterine, fetal, and placental activities. These include emmenagogues, ecbolic drugs, contraceptives, uterine sedatives for females, and depurate or drugs that hamper male sexual and reproductive capabilities, affect fluidity or motility of the seminal fluid, destroy sperms, or impede libido.

A large number of drugs are known to have sterilizing, contraceptive, and abortifacients properties. However, these indigenous means and drugs were extensively used even in rural or tribal cultures until the 20th century, when there has been no noteworthy systematic or scientific efforts to study these aspects except for a few intermittent studies. While the list of such ingredients is quite big, unusually small scientific data are available about the nature of their active components and about their mechanisms of action.

As biotechnology-based advances open up new vistas in biomedical research, it will be of interest to examine the subject of contraception once again, as in Ayurveda, in the light of present-day pharmacology for future possibilities.

A thoughtful attempt has been made here to explore Ayurvedic and scientific aspects of formulations and ingredients as described in multiplicity of classical texts covering different facets of contraception.

## Methodology

Ancient classical texts, medieval compendia, and other pertinent texts were assessed for enlisting different methods used for contraception and to enlist formulations and ingredients used for a variety of activities that could be pharmacologically linked to contraception. Specific search was undertaken for any existing review that could add to information on the subject. A systematic review of published articles on the subjects related to contraception was undertaken. The description of methods used in the experimental animal models, and the antifertility effect of active ingredients, their doses, safety, and toxicity were examined. Ninety-four plants and six minerals are reported in this review having a variety of contraceptive activities.

**FIGURE 2:**
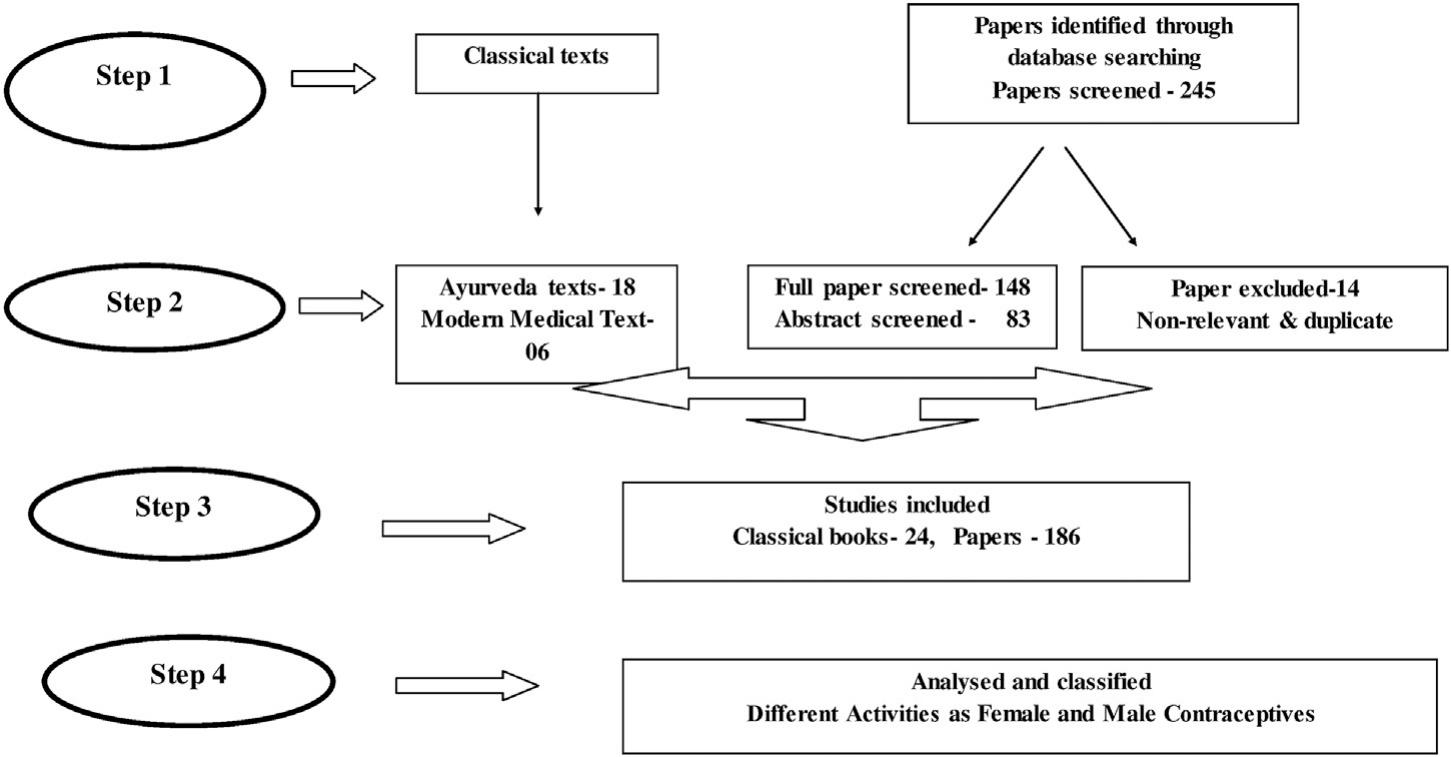
Flowchart of the systemic review process searching for contraceptive plants.

Flowchart of the systematic review process to search for contraceptive plants is presented. (Figure 2).

## Contraceptives in Ayurveda and Medieval Sanskrit Literature

Ayurvedic literature is rife with thousands of formulations and has about 1100 ingredients attributed with well-defined therapeutic approaches including reproduction. There are references to temporary or permanent sterilization. Search for contraception from traditional knowledge of Ayurveda has been of interest to the Central Drug Research Institute, Council of Scientific and Industrial Research under Ministry of Science and Technology, and the Central Council for Research in Ayurvedic Sciences under Ministry of Health (now Ministry of AYUSH), bodies under the Government of India. Several other private and industry organizations had undertaken studies in the past. However, there is a need to revive research interest in Ayurveda in reproductive biology for safe, low-cost, user-friendly, and reliable therapeutic solutions to satisfy different contraception requirements.

### Vedic Period (1500-500 BCE)

Regulated sexual life or abstinence from sex was considered the ideal method of contraception in *Vedic* times. The emphasis was more on propensity of the right, healthy progeny. Indirect references to contraception can be found in the *Atharva Veda.*

The use of drugs leading to impotence as punishment meted out to a person committing social sins or to an enemy or infliction of injury to two cords situated near the scrotum or the scrotum itself, to put an end to one’s desire for progeny were in practice. These can be considered as references for use of drugs to prevent conception, vasectomy, and castration, respectively (Satvalekar, 1958a).

A mechanical device made of stone to obstruct multiple channels of *Yoni—*the vaginal cavity to prevent conception has been mentioned. This could be considered as the earliest form of an intrauterine contraceptive device. Similarly, artificially induced changes to make the vaginal cavity rough or dry, besides its mechanical obstruction for futile coitus have been mentioned (Satvalekar, 1958b). This reference reflects some chemical changes to be produced artificially, probably in the cervical mucus obstructing the entry of sperms, or in the endometrium influencing the implantation of the zygote and a mechanical barrier in the vaginal canal. (Tewari and Chaturvedi, 1981). In *Brhadaranyaka Upanishad*, a breath exercise is advised during coitus to avoid conception (Dash and Basu, 1968).

### Samhita Period: (300-500 BCE)

Though *Charak Samhita, Sushrut Samhita*, and *Ashtang Sangraha*–*Bruhatryee,* the three ancient most Ayurveda treatises, have elaborated the subject of reproduction extensively, there are no direct references to contraception.

*Kshetra*—the female reproductive system as the field, *ambu—*the nutrient fluids, *bija*—the sperm or ovum as the seed, *rutukal*—the ideal ovulatory period, *marga—*the female canal*, Vayu—the* neural system, and *hrid*—the psychological status are considered the essential factors for conception. Any or more of these factors if influenced artificially can lead to a failure of conception. The *shukravaha srotas* and *aartavavaha srotas* representing seminal and menstrual flows, respectively, are among the 13th intrinsic and interdependent biological pathways or channels (that could be explained based on now prevalent means of system biology). This early knowledge could pave the way for the development of different kinds of contraceptive methods prevailing in the present scenario, and all of them influence one or the other factors that have been explained in the ancient classics (Vagbhatt, 2000; Sushrut, 2002).

**FIGURE 3:**
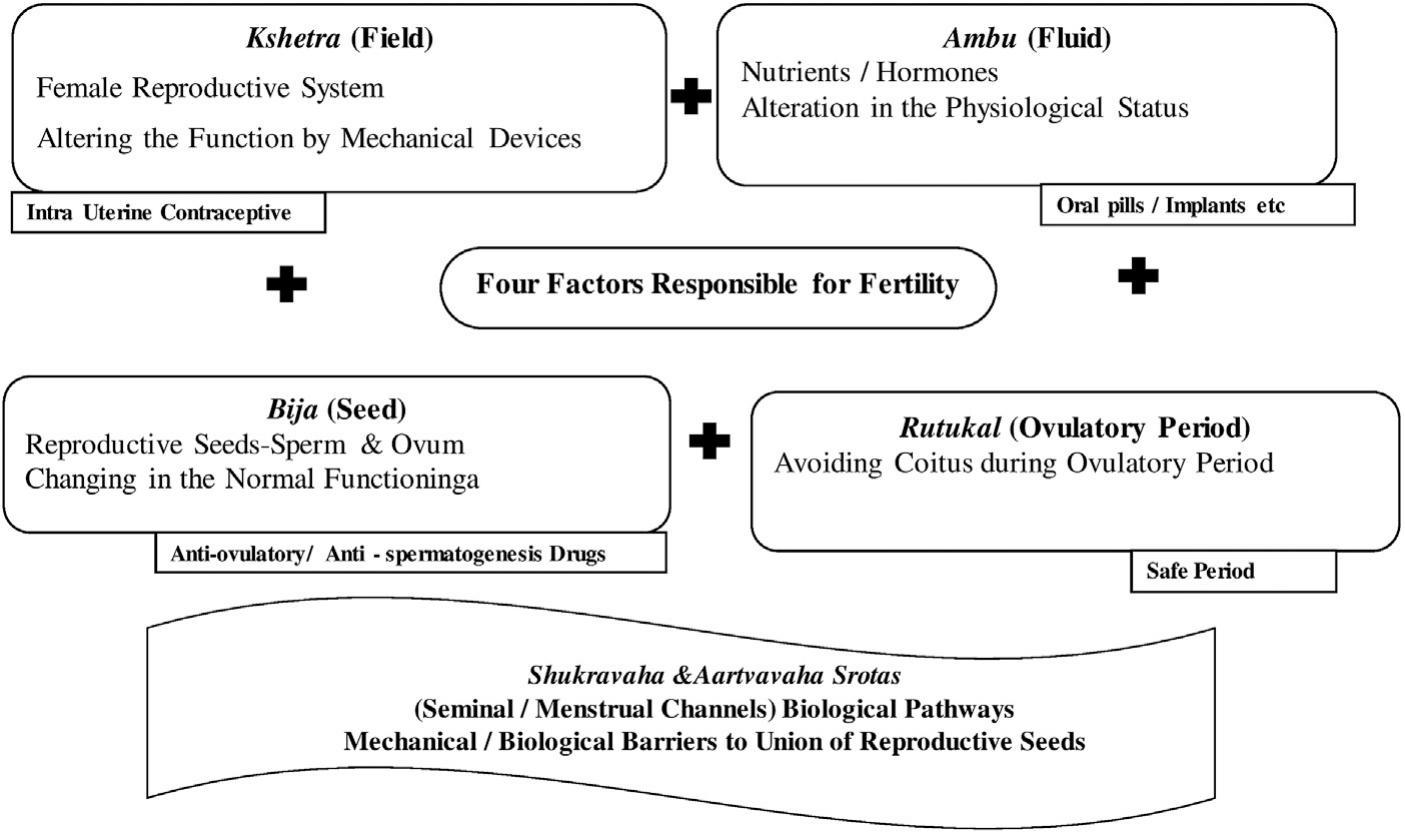
Contraception in the context of Ayurvedic principle of fertility.

Contraceptive activities in the context of Ayurvedic principle of fertility are explained in Figure 3 *.*

### Medieval Period (1000 AD to 1900 AD)

*Rajamartanda* written in the 11th century is probably one of the earliest texts to mention a specific prescription for contraceptives. Compendia texts like *Bhava Prakash*, *Yoga Ratnakara*, *Bhaishajya Ratnavali*, *Gadanigrah*, and several others prescribe many herbal and herbo-mineral contraceptive preparations for local and oral use by men and women.

By the 11th century, the oriental connectivity that had sociocultural effects also brought in practices to prevent conception or induce abortion. References to oral and local contraceptives are found in *Bruhad Yoga Tarangini* and *RatiRahasya* [AD800], *RasaPrakashSudhakar* [AD1300], *Panchasayaka, Smaradeepika and RasaRatnaSamuccchay* [AD1400], *RatiManjiri* [AD1500], *Kandarpchudamani* [AD1577], *AnnangaRang, Bhavprakash and YogaRatnakar* [AD1600], *YogaRatnaSamucchaya* [AD 1800], *and Brihan Nighantu Ratnakar and BhaishjyaRatnaval i*[AD 1900].

The subject of contraceptives in ancient times dealt not only with medieval medicine but also with art and the literary works of poets, playwrights, and philosophers. Like *Kama Sutra*, the famous text on erotica, a large number of books in the 19th century contain various recipes for contraception and for inducing abortions and diverse birth control practices.

#### Some of the most prescribed practices and recipes for preventing conception are as follows.

1. Local Contraceptives for Females
  Vaginal fumigation or application before coitus with (1) moistened *Saindhava lavana (Rock salt)* with *Til (Sesame) oil.* (Jugnu and Sharma, 2011), (2) wood of *Neem (Azadirachta indica* A. Juss. ) before coitus (Tripathi, 1969), and (3) powdered root of *Dhattura (Datura metel* L*.)* plucked on the 14th°day (dark night) of the lunar month[Indradev, 1998] or tying the waist with roots (Lakmipatishashtri, 1983).
2. Oral Contraceptives
  • Powder of *Pippali (Piper longum* L*.) and Vidanga (Embelia ribes* Burm.f.) with *Tankana (Borax)* taken in equal quantity in fertile phase with milk (Lakmipatishashtri, 1983).
  • Flowers of *Japa (Hibiscus rosa-sinensis* L.*)*: immediately after the delivery of a child (Bhavamisra, 1961; Lakmipatishashtri, 1983) or with *Kanji (fermented drink)* along with 48 grams of old jaggery to be taken for 3°days in the fertile phase. (Lakmipatishashtri, 1983).
  • Root of *Tanduliyaka (Amaranthus spinosus* L.*)* with *Tandulodaka (rice water)* to be taken after menstruation for 3°days. (Lakmipatishashtri, 1983).
  • Powders of *Talisa patra (Abies spectabilis* (D.Don) Mirb.) and *Gairika (Red Ochre, Fe*_*2*_ *O*_*3*_) in equal parts to be consumed on the 4th day of menstruation with water. (Lakmipatishashtri, 1983).
  • Aqueous extract of *Rasanjana (Extract of Berberis aristata* DC.*)*, *Hemavati (Sweta – Vacha)* (*Iris × germanica* L.), and *Vayastha (Terminalia chebula Retz.)* with cold water. (Rajeshwaradatta, 2001).
  • Powders of *Amla* (*Phyllanthus emblica* L.), *Arjuna* (Terminalia arjuna (Roxb. ex DC)), and *Abhaya (Terminalia chebula Retz.)* with water. (Rajeshwaradatta, 2001).
  • Paste made of the root of *Chitraka (Plumbago zeylanica* L.) mixed with *Nirgundi (Vitex negundo* L.*)* juice given orally in the dose of one 12°gm with honey. (Lakmipatishashtri, 1983).
  • Powder of seeds of *Sarshapa* (*Brassica rapa* L.) with *Tanduliyam* (*Amaranthus spinosus* L*.*) and *Sarkara* (*Sugar candy*) pounded with *Tandulodaka* (*rice water*) given with milk. (Jugnu and Sharma, 2011).
  • Ashes of *Sehund stem (Euphorbia neriifolia* L*.),* 12°g daily. (Kuchimara, 2007).
  • *Rhizome of Haridra (Curcuma longa* L.*)* daily during the 3°days of menstruation followed by an additional 3°days (Kuchimara, 2007).
  • Powders of *Krishna Jeeraka (Carum carvi* L.*), Karchooram (Hedychium spicatum* Sm*.), Nagakesara (Mesua ferrea L.), Haritaki (Terminalia chebula* Retz.*), Kalonji (Nigella sativa* L*.),* and *Kayaphala (Myrica nagi* Thunb.*)* made into pills in the size of ziziphus fruit for 7°days. (Kuchimara, 2007).
3. Abortifacient
  • Root of Sweta Aparajita (*Clitoria ternatea* L.), Kakadani (Sarngesta)(*Cardiospermum halicacabum* L.) or Punarnava (*Boerhavia diffusa* L.) with oil of Eranda (*Ricinus communis* L.)—Patradanda (stem of leaf) to be inserted in the vagina (Rajamartanda), 1966; Tripathi, 1969; Lakmipatishashtri, 1983
  • Devalaya Churna (scrapped lime powder from the wall of temple) 12°g with water. (Lakmipatishashtri, 1983; Indradev, 1998).
  • Seeds of Grnjana (Carrot) (*Daucus carota* L) with roots of Tuvari (*Cajanus cajan* (L.) Huth) and Sindura (lead oxide).
  • Ghotipurisa (feces of mare) mixed with Kanji, filtered, and mixed with rock salt, Ugra (*Apium graveolens* L.), and AsuriTaila (Oil of *Brassica juncea* (L.) Czern.) with Visha (*Aconitum chasmanthum* Stapf ex Holmes) (Lakmipatishashtri, 1983).

Plant and mineral drugs mentioned as contraceptives in the Ayurvedic classical texts are given in Table 1.

**TABLE 1:** List of plant and metal drugs as contraceptives in Ayurveda classics. Vertical column numbers indicate *AaartavJanan*—Emmenagogue (1), *Aparapatan*—placental expulsion (2), *Garbhanuloman/Garbhapatkar*—Abortifacient or *Garbhastravakar*—expel Fetus (3), *Garbhanirodhak Contraceptives* (4), *Garbhashayasancochak—*Ecbolic (5), *Shandhyakar/Pumstvopadhatin*— drugs that hamper male sexual or reproductive capability (6), and *Shukrashodhan*—Depurates (7).

Sr. No.	Sanskrit name	Botanical name	(1)	(2)	(3)	(4)	(5)	(6)	(7)	
	*Aguru*	*Aquilaria malaccensis* Lam.		√						
	*Ahiphen*	Papaver somniferum L.						√		
	*Amalaki*	*Phyllanthus emblica L*				√				
	*Ashok*	*Saraca asoca* (Roxb.) J.J.de Wilde	√				√			
	*Asuri*	*Brassica juncea (L.) Czern*			√					
	*Arjuna*	*Terminalia arjuna* (Roxb. ex DC.) Wight &Arn.				√				
	*Bhanga*	*Cannabis sativa* L.						√	√	
	*Bhurjapatra*	*Betula utilis* D. Don		√	√					
	*Chandan*	*Santalum album* L.						√		
	*Chavya*	*Piper retrofractum* Vahl		√						
	*Chirbilva*	*Holoptelea integrifolia (Roxb.) Planch*.			√					
	*Chitraka*	*Plumbago zeylanica* L.	√	√	√	√				
	*Chuka*	Rumex acetosa L.						√		
	*Devdaru*	*Cedrus deodara* (Roxb. ex D. Don) G. Don	√	√						
	*Dhanyak*	*Coriandrum sativum* L.						√		
	*Dhattura*	*Datura metel* L.				√		√		
	*Ela*	*Elettaria cardamomum* (L.) Maton		√	√					
	*Eranda*	*Ricinus communis* L.			√					
	*Eshvari*	*Aristolochia indica* L.			√		√			
	*Grnjana*	*Daucus carota* L.			√					
	*Haridra*	*Curcuma longa* L.				√				
	*Haritaki*	*Terminalia chebula* Retz.				√				
	*Harmal*	*Peganum harmala* L.					√			
	*Hemavati*	*Iris germanica* L.				√				
	*Hingu*	*Ferula assa-foetida L.*			√					
	*Hirabol*	*Commiphora myrrha* (Nees) Engl.					√			
	*Japa*	*Hibiscus rosa-sinensis* L.				√				
	*Karchuram*	*Hedychium spicatum* Sm.				√				
	*Kadamb*	*Neolamarckia cadamba* (Roxb.) Bosser							√	
	*Kakadani (Sarngesta)*	*Cardiospermum halicacabum* L.			√					
	*Kakamachi*	*Solanum nigrum* L.						√		
	*Karpas*	*Gossypium herbaceum* L.	√				√			
	*Karpur*	*Cinnamomum camphora (*L.) J. Presl						√		
	*Kasani*	*Cichorium intybus* L.						√		
	*Kayaphala*	*Myrica nagi* Thunb.				√			√	
	*Ketaki*	*Pandanus tectorius* Parkinson ex Du Roi			√					
	*Krishna Jeeraka*	*Carum carvi* L.			√	√				
	*Kulattha*	*Vigna unguiculata* (L.) Walp.	√	√				√		
	*Kushtha*	*Aucklandia costus* Falc	√	√	√				√	
	*Langali*	*Gloriosa superba* L*.*			√		√			
	*Lodhra*	*Symplocos racemosa* Roxb.					√			
	*Mandukparni*	*Centella asiatica* (L.) Urb.		√						
	*Mocharas*	*Bombax ceiba* L.					√			
	*Nagakesara*	*Mesua ferrea* L.				√				
	*Nagdamani*	*Artemisia nilagirica* (C. B. Clarke) Pamp.			√					
	*Neem*	*Azadirachta indica* A. Juss.				√	√			
	*Nimbu*	Citrus × aurantium L.						√		
	*Nilophar*	*Nymphaea alba* L.						√		
	*Nirgundi*	*Vitex negundo* L.			√	√		√		
	*Pippali*	*Piper longum* L.		√		√				
	*Punarnava*	*Boerhavia diffusa* L			√					
	*Rasanjana*	*Berberis aristata* DC.				√				
	*Rason*	*Allium cepa* L.						√		
	*Sarshapa*	*Brassica rapa* L.					√			
	*Sehund*	*Euphorbia neriifolia* L.					√			
	*Shal-sarjarasa*	*Shorea robusta* Gaertn.	√							
	*Shallaki*	*Boswellia serrata* Roxb.					√			
	*Shan*	*Dioscorea polystachya* Turcz.	√							
	*Shigru*	*Moringa oleifera* Lam.			√					
	*Shinshapa*	*Dalbergia sissoo* Roxb. ex DC.			√					
	*Shyonak*	*Oroxylum indicum* (L.) Kurz					√			
	*Sitab*	*Ruta graveolens* L.					√	√	√	
	*Sitaphal*	*Annona squamosa* L.			√	√				
	*Sunthi*	*Zingiber officinale* Roscoe		√						
	*Sweta Aparajita*	*Clitoria ternatea* L.			√					
	*Talisa patra*	*Abies spectabilis (D. Don) Mirb.*	√	√		√				
	*Tanduliyaka*	*Amaranthus spinosus* L.				√				
	*Tintidika*	*Tamarindus indica* L.						√		
	*Tilataila*	*Sesame oil*				√				
	*Tuvari*	*Cajanus cajan* (L.) Huth			√					
	*Ugra*	*Apium graveolens* L.			√					
	*Ulatakambal*	*Abroma augusta* (L.) L.f.	√				√			
	*Unnab*	*Ziziphus jujuba* Mill.						√		
	*Upakunchika*	*Nigella sativa L.*		√		√	√			
	*Ushir*	*Chrysopogon zizanioides* (L.) Roberty							√	
	*Vacha*	*Acorus calamus* L.			√					
	*Vansha*	*Bambusa bambos* (L.) Voss	√					√		
	*Vidanga*	*Embelia ribes* Burm.f.	√	√		√				
	*Visha*	*Aconitum chasmanthum* Stapf ex Holmes			√					
Minerals/Metals										
	*Devalaya Churna*	Scrapped lime powder from the wall of temple				√				
	*Gairika*	*Red Ochre, Fe*_*2*_ *O*_*3*_				√				
	*Nausagar*	NH_4_Cl						√		
	*Saindhava lavana*	*Rock salt*				√				
	*Sindura*	*Lead oxide*			√	√				
	*Tankana*	*Borax*				√				

It is observed that 79 plant drugs and six mineral drugs are used as abortifacients, oral contraceptives, or as local applications along with *Kanji* (fermented drink), *Tandulodaka* (rice water), *Sarkara* (sugar candy), milk, and honey.

## Potential Ingredients Having Antifertility or Contraceptive Properties

This literature survey revealed that there are about more than 94 indigenous medicinal plants having scientific evidence of acting as contraceptives. Some of the remarkable plant drugs with parts used, their chemical constituents, and pharmacological activities are described in Table 2. This compiled information will provide useful reference for new drug designing models, acting either as male or female contraceptives.

**TABLE 2:** Medicinal plants and their phyotoconstituents validated for various female/male contraceptive activities. Different contraceptive activities studied on medicinal plants could be categorized as follows. Female contraceptive activities: (2A) anti-implantation activity, (2B) abortification, (2C) antifertility, (2D) antiovulatory, and (2E) antiestrogenic activity. Male contraceptive activities: (2F) antispermatogenic, (2G) spermicidal, and (2H) antiandrogenic activity.

Sr. No.	Botanical name, family, Sanskrit name, parts	Chemical composition	Extract	Mode of action in experimental studies	Reference
A Anti-implantation activity					
1.	*Abies spectabilis* (D. Don) Mirb.	Flavonoids, bioflavonoids, glycosides, phytosterols	Benzene, alcoholic	Anti-implantation activity	Anonymous (1996)
	Pinaceae				
	Talisa Patra, leaf				
2.	*Abroma augusta* (L.) L.f.	L-rhamnose, L-arabinose, D-xylose, D-mannose, D-galactose, D-glucose, D-galacturonic acid, and D-glucuronic acid	Alcoholic	Anti-implantation	Maurya et al. (2004), Pokharkar et al. (2010), Kalita et al. (2011)
	Malvaceae				
	Pishach karpas, roots				
3.	*Adhatoda vasica* Nees synonym of *Justicia adhatoda* L. Acanthaceae	Alkaloids, tannins, saponins, and phenolics flavonoids	Aqueous	Anti-implantation	Pokharkar et al. (2010); Kaur et al. (2011); Raj et al. (2011)
	Vasa, leaves				
4.	*Ailanthus excelsa* Roxb	Sitosterol, quassinoids, and ailantic acid	Ethanolic	Anti-implantation decreased of implant sites	Priya et al. (2012); Tamboli and Konadawar (2013)
	Simaroubaceae				
	Maharukha, leaves				
5.	*Allium cepa* L.	Kampferol, β-sitosterol, ferulic acid, and myritic acid	Ethanolic	Anti-implantation inhibition of implant sites	Thakare et al. (2009); Ola-Mudathir et al. (2008)
	Amaryllidaceae				
	Palandu, onion, bulb				
6.	*Aloe barbadensis* Mill.	Water, polysaccharides, pectin, cellulose, hemicellulose, and glucomannan	Ethanolic and aqueous	Anti-implantation	Shah et al. (2017), Shah et al. (2016)
	Synonym of aloe vera (L.) Burm.f.				
	Asphodelaceae				
	Kumari, leaves				
7.	*Areca catechu* L.	Alkaloids—pilocarpine, arecaidine, and arecoline	Petroleum ether, alcoholic, and aqueous	Anti-implantation	Garg and Garg (1970); Garg and Garg (1971)
	Arecaceae				
	Poogaphala, Nuts				
8.	*Cassia fistula* L.	Alkaloid	Aqueous	Anti-implantation, decreased glycogen content in uterus, and antifertility	Yadav and Jain (2009)
	Fabaceae				
	Aragvadha, fruits, bark				
9.	*Carica papaya* L.	Papain, caricacin, carpasemine, and oleanolic glycoside	Pet ether, alcohol, and aqueous ethanol	60 % anti-implantation activity, abortifacient in albino rats	Garg and Garg (1970); Garg and Garg (1971); Das (1980); Sinha and Nathawat (1989); Changamma and Lakshman (2013)
	Caricaceae,				
	Papaya unripe fruit pulp, seeds, latex				
10.	*Centratherum anthelminticum* (L.) Gamble	Glycosides, carbohydrates, phenolic compounds, tannins, flavonoids, proteins, saponins, and sterols	Ethanol	Postcoital anti-implantation activity	Sharma et al. (1994)
	Asteraceae				
	Vanya Jeeraka, seeds				
11.	*Citrus × aurantium* L	Citroflavanoids, glucosides, and triterpenoids	Petroleum ether	Anti-implantation, antiovulatory, abortifacients increased ovarian weight, decreased Graafian follicles, and irregular estrous cycle	Patil and Patil (2013)
	Rutaceae				
	Bijaura, seeds				
12.	*Embelia ribes* Burm.f.	Embelin, volatile oil, and fixed oil	Isolated embelin	Anti-implantation and postcoital antifertility activity	Prakash (1981); Nand (1981); Dixit and Joshi (1983)
	Primulaceae				
	Vidang, berries				
13.	*Gloriosa superba* L.	Colchicine (superbine)	Hydroalcoholic extract at two different doses	Antifertility, anti-implantation activity in postcoital study, abortifacient activity	Latha et al. (2013)
	Colchicaceae				
	Langli				
	Root				
14.	*Grewia asiatica* L.	Potassium, calcium, phosphorus, copper, zinc, and magnesium	Aqueous	Anti-implantation and abortification activity	Kamboj and Dhawan (1982)
	Malvaceae, seeds				
15.	*Hibiscus rosa-sinensis* L.	Cyclopeptide alkaloid	Ethanol and benzene extract	Anti-implantation, antiovulatory, increased uterine weight, secretion of estrogenic by atretic follicles, postcoital antifertility	Neeru and Sharma (2008); Vasudeva and Sharma (2008); Hadimur et al. (2014), Pal et al. (1985)
	Malvaceae				
	Japa				
	Flowers				
16.	*Mesua ferrea* L.	Mesuol, mammegin, mesuaferronea, and mammeuisin	Aqueous	Anti-implantation activity	Seshadri and Pillai (1981); Munshi et al. (1977)
	Calophyllaceae				
	Nagakeshara, flowers				
17.	*Michelia champaca* L.	Essential oil	Benzene and hydroalcholic extract	Postcoital anti-implantation activity	Sharma et al. (1994); Taprial et al. (2013)
	Magnoliaceae				
	Champaka, Anthers				
18.	*Momordica charantia* L.	Glycosides, saponins, alkaloids, fixed oils, triterpenes, proteins, and steroids	Aqueous	Uterine stimulant activity, Antifertility, estrogenic activity	Jamwal and Anand (1962); Saksena (1971)
	Cucurbitaceae				
	Karwellaka				
	roots, leaves				
19.	*Plumbago zeylanica* L.	Plumbagin	Plumbagin-free alcohol	Anti-implantation and abortifacient activity	Gupta et al. (2011)
	Plumbaginaceae				
	Chitrak, root				
20.	*Ricinus communis* L.	Ricinine and isoquinoline	Aqueous	Anti-implantation, increase in diameter of the uterus, and decrease in uterine hormones	Makonnen et al. (1999)
	Euphorbiaceae				
	Erand, castor bean				
	Seed				
21.	*Rubia cordifolia* L.	Munjistin, purpurin, and pseudopurpurin	Ethanolic extract	Anti-implantation	Maurya et al. (2004)
	Rubiaceae				
	Manjishtha				
	Root				
22.	*Sapindus trifoliatus* L.	Essential oil	Butanol	Antizygotic, blastocytotoxic, or anti-implantation activity	Pal et al. (2013); Bodhankar et al. (1974)
	Sapindaceae				
	Arishtak				
	Fruits, pulp, and seeds				
23.	*Sesbania sesban* (L.) Merr. Fabaceae	Alkaloids, flavonoids, glycosides, tannin, anthraquinone, steroid, pholobatannins, and terpenoids	Extract and powder	Inhibit the ovarian function, change the uterine structure, and prevent the implantation	Singh (1990a); Samajdar and Ghosh (2017)
	Sesban				
	Leaves				
B Abortification activity					
1.	*Abroma augusta* (L.) L.f.	L-rhamnose, L-arabinose, D-xylose, D-mannose, D-galactose, D-glucose, D-galacturonic acid, and D-glucuronic acid	Alcoholic	Abortification activity	Pokharkar et al. (2010); Kalita et al. (2011)
	Malvaceae				
	*Pishach karpas,* roots				
2.	*Abrus precatorius* L.	Abrin, abrasine, precasine, and precol	Aqueous	Abortifacient activity or antifertility agent with a risk of DNA damage	Sarwat et al. (2009); Kaur et al. (2011); Shrivastava et al. (2007); Azmeera et al. (2012); Priya et al. (2012)
	Papilionaceae				
	*Gunja,* Seeds				
3.	*Achyranthes aspera* L.	Fatty acids, oleonic acid, bisdesmosidic, triterpenoid alkaloids, D-glucuronic, betaine, and achyranthine	Benzene, ethanolic, and chloroform	Abortifacient activity in rabbits	Raj et al. (2011); Vasudeva and Sharma (2006)
	Amaranthaceae				
	*Apamarga*Whole plant,				
	Stem bark, Root				
4.	*Adhatoda vasica* Nees synonym *of Justicia adhatoda* L.	Alkaloids, tannins, saponins, phenolics, and flavonoids	Aqueous	Abortification activity	Pokharkar et al. (2010); Kaur et al. (2011); Raj et al. (2011)
	Acanthaceae*, Vasa,* Leaves				
5.	*Aegle marmelos* (L.) Corrêa*.*	Marmelosin, luvangetin, psoralen, tannins, and marmin	Aqueous extract	Abortifacient activity in albino rats	Gangadhar and Lalithakumari (1995); Sathiyaraj et al. (2010)
	Rutaceae				
	*Bilva,* whole plant, leaves				
6.	*Annona squamosa* L.	Atropine alkaloids, and anonaine	Ethyl acetate extract	Abortifacient induces early abortion	Jain and Dixit (1992)
	Annonaceae				
	*Custard apple*				
	Seeds, leaves, and bark				
7.	*Areca catechu* L.	Alkaloids—pilocarpine, arecaidine, and arecoline	Petroleum ether, alcoholic, and aqueous	Abortifacient activity in albino rats and antifertility activity	Garg and Garg (1970); Garg and Garg (1971); Shrestha et al. (2010)
	Arecaceae				
	*Poogaphala,* nuts				
8.	*Barleria prionitis* L.	Acbarlerin, barlerin, ß-sitosterol, flavanol glycoside, and iridoids	Methanol extract	Abortifacient	Gupta et al. (2000)
	Acanthaceae				
	*Saireyak,* Roots				
9.	*Carica papaya* L.	Papain, caricacin, carpasemine, and oleanolic glycoside,	Pet ether, alcohol, and aqueous ethanol	Abortifacient in albino rats and antifertility	Garg and Garg (1970), Garg and Garg (1971); Das (1980); Sinha and Nathawat (1989); Changamma and Lakshman (2013)
	Caricaceae				
	Papaya unripe fruit pulp,				
	seeds, and latex				
10.	*Citrus × aurantium* L	Citroflavanoids, glucosides, and triterpenoids	Petroleum ether	Abortifacient, increased ovarian weight, decreased Graafian follicles, and irregular estrous cycle	Patil and Patil (2013)
	Rutaceae				
	*Bijaura,* Seeds				
11.	*Daucus carota* L.	Essential oil	Petroleum, ether, benzene, alcohol, and water	Abortifacient activity	Garg (1975); Jansen and Wolhlmuth (2014); Shah and Varute (1980)
	Apiaceae				
	*Grinjanak,* seed				
12.	*Gloriosa superba* L.	Carbohydrates, flavonoids, steroids, alkaloids, tannins, and glycosides	Ether, chloroform, and ethyl alcohol extracts	Abortifacient activity and significant reduction in number of implants and number of pups born	Malpani and Mahurkar (2018)
	Colchicaceae				
	*Langli*				
	Root				
13.	*Grewia asiatica* L.	Potassium, calcium, phosphorus, copper, zinc, and magnesium	Aqueous	Abortification activity	Kamboj and Dhawan (1982)
	Malvaceae, seeds				
14.	*Lepidium sativum* L.	Lepidine	Methanolic	Abortifacient and antiovulatory	Pande et al. (2002)
	Brassicaceae				
	*Chandrasur*				
	Mature explants				
15.	*Ricinus communis* L.	Ricinine and isoquinoline	Aqueous extract	Abortifacient	Makonnen et al. (1999), Sandhyakumary et al. (2003)
	Euphorbiaceae				
	*Erand, Castor bean*				
	Seed				
16.	*Woodfordia fruticosa* (L.) Kurz	Tannins, flavonoids, anthraquinone glycosides, and polyphenols	Aqueous and ethanol	Abortifacient	Pathak et al. (2005)
	Lythraceae				
	*Dhataki,* flowers				
C Antifertility activity					
1.	*Abrus precatorius* L.	Abrin, abrasine, precasine, and precol	Aqueous	Antifertility agent with a risk of DNA damage	Sarwat et al. (2009); Kaur et al. (2011); Shrivastava et al. (2007); Azmeera et al. (2012); Priya et al. (2012)
	Papilionaceae				
	*Gunja,* Seeds				
2.	*Acacia leucophloea* (Roxb.) Willd.	N-hexacosanol, beta-amyrin, beta-sitosterol, and tannin	Alcoholic	Antifertility activity	Dheeraj (2011)
	Leguminosae—Fabaceae				
	*Shwet babul,* roots				
3.	*Annona squamosa* L.	Atropine alkaloids and anonaine	Ethyl acetate extract	Abortifacient—induces early abortion	Jain and Dixit (1992)
	Annonaceae				
	*Custard apple*				
	Seeds, leaves, and bark				
4.	*Areca catechu* L.	Alkaloids—pilocarpine, arecaidine, and arecoline	Nut oil Ethanolic extract	Antifertility activity in female albino rats, antiovulatory, and ovarian weight decreased due to imbalance in gonadotrophins	Garg et al. (1974); Shrestha et al. (2010)
	Arecaceae				
	*Poogaphala,* Nuts				
5.	*Azadirachta indica* A. Juss	Azadirachtin, nimbolinin, nimbin, nimbidin, nimbidol, sodium nimbinate, and gedunin	Female albino rabbits Seed oil	Antifertility and functional sterility	Vyas and Purohit (2018)
	Meliaceae				
	*Nimba*				
	Leaves, flower, and seed				
6.	*Carica papaya* L.	Papain, caricacin, carpasemine, and oleanolic glycoside	Pet ether, alcohol, aqueous, and ethanol	Antifertility	Garg and Garg (1970); Garg and Garg (1971); Das (1980); Sinha and Nathawat (1989); Changamma and Lakshman (2013)
	Caricaceae				
	Papaya unripe fruit pulp,				
	seeds, and latex				
7.	*Cissampelos pareira* L.	Berberine	Leaf extract	Altered the estrous cycle pattern in female mice, Antifertility	Ganguly et al. (2007); Samatha et al. (2011)
	Menispermaceae*, Patha*				
	Leaves and stem				
8.	*Cuminum cyminum* L*.*	Cuminal and cuminic alcohol	Extract	Antifertility effect in female albino rat	Priya et al. (2012); Sharma J et al. (2001)
	Apiaceae				
	*Jeerak,* seeds				
9.	*Crateva nurvala* Buch-Ham.	Alkaloids, triterpene, tannins, saponins, flavonoids, sterols, glucosylinate, lupeol, and diosgenin	Ethanol, aqueous	Antifertility effects estrogenic activity	Bhaskar et al. (2009)
	Capparaceae				
	*Varuna*				
	Dried stem bark				
10.	*Curcuma longa* L.	Curcumin and flavanoids	Ethanol, aqueous	Propylene glycol solution, antifertility, antiovulatory—suppression of GnRH	Ghosh et al. (2011); Bhagat and Purohit (1986)
	Zingiberaceae				
	*Haldi,* rhizome				
11.	*Daucus carota* L.	Essential oil	Petroleum, ether, benzene, alcohol, and water	Antifertility activity	Garg (1975); Jansen and Wolhlmuth (2014); Shah and Varute (1980)
	Apiaceae				
	*Grinjanak,* Seed				
12.	*Desmodium gangeticum* (L.) DC.	Lavonoid glycosides, pterocarpanoids, lipids, glycolipids, and alkaloids	Gangeticum	Antifertility effect	Pillai et al. (1982)
	Fabaceae				
	*Shaliparni,* Root				
13.	*Embelia ribes* Burm.f.	Embelin, volatile oil, fixed oil, resin, tannin, christembine (alkaloid), and phenolic acids	Isolated embelin	Anti-implantation and postcoital antifertility activity	Prakash (1981b)
	Primulaceae				
	*Vidang,* Berries				
14.	*Ferula jaeschkeana Vatke*	Flavonoids, alkaloids, terpenoids, cardiac glycosides, saponins, and phenolics	Hexane	Duration-dependent luteolytic changes in the corpora lutea	Pathak et al. (1995)
	Apiaceae				
	*Heengupatri,*				
	Dried leaves				
15.	*Gloriosa superba* L.	Colchicine (superbine)	Hydroalcoholic extract at two different doses 30 and 60°mg/kg	Antifertility, anti-implantation activity in postcoital study	Latha et al. (2013)
	Colchicaceae				
	*Langli,* Root				
16.	*Hibiscus rosa-sinensis* L.	Cyclopeptide alkaloid	Ethanol and benzene extract	Anti-implantation, antiovulatory, secretion of estrogenic by atretic follicles, and postcoital antifertility	Neeru and Sharma (2008)
	Malvaceae				
	*Japa*				
	Flowers				
17.	*Lawsonia inermis* L.	Lawsone, esculetin, fraxetin, isoplumbagin, scopoletin, betulin, betulinic acid, hennadiol, lupeol, lacoumarin, quinone, and napthaquinone	Powder	Preventing pregnancy in 60% of the animals tested	Munshi et al. (1977)
	Lythraceae				
	*Madayantika*				
	Leaves				
18.	*Lepidium sativum* L.	Lepidine	Methanolic	Abortifacient and antiovulatory	Pande et al. (2002)
	Brassicaceae				
	*Chandrasur*				
	Mature explants				
19.	*Melia azedarach* L. *Meliaceae, MalaiVembu*	Triterpenoids	Seed extract	Antifertility effect, increased preimplantation, postimplantation, and total prenatal mortalities	Mandal and Dhariwal (2007)
	seed and leaves				
20.	*Momordica charantia* L.	Glycosides, saponins, alkaloids, fixed oils, triterpenes, proteins, and steroids	Aqueous	Uterine stimulant activity, antifertility, and estrogenic activity	Jamwal and Anand (1962); Saksena (1971)
	Cucurbitaceae				
	*Karwellaka*				
	Roots and leaves				
21.	*Nigella sativa* L.	Fixed oil, volatile oil, and alkaloids	Hexane	Antifertility activity in rats, postcoital contraceptive	Keshri et al. (1995)
	Ranunculaceae				
	*Krishna jeerak,* Seeds				
22.	*Piper betle* L*.*	Eugenol, eugenol acetate, piper betol, piperol, and methyl eugenol phytol	Alcoholic	Antifertility, antiestrogenic effects in female rats	Sharma et al. (2007)
	Piperaceae				
	*Betel leaf, Pan*				
	Petiol				
23.	*Piper longum* L.	Piperine	Powder, hexane fraction, and benzene	Antifertility activity—prolonged the length of the extort cycle, drastic reduction in the number of implantation sites, marked suppression in the ovarian cytokines and nitric acid level	Laxmi et al. (2006); Kholkute et al. (1979)
	Piperaceae				
	*Pippali*				
	Root and ruits				
24.	*Trichosanthes cucumerina* L.	Cucurbitacin B, cucurbitacin E, isocucurbitacin B, E, sterols 2 β-sitosterol stigmasterol	Aqueous	Affected the normal estrous cycle, significantly reduced the number of healthy follicles, corpora lutea, and increased the number of regressing follicles. Reduced serum FSH and LH levels	Devendra et al. (2009)
	Cucurbitaceae				
	*Snake gourd,* Fruit				
25.	*Zingiber officinale*	Monocyclic, phenols, sesquiterpenees sential oil, oleoresins, and proteolytic enzymes	Aqueous, ethanol extracts	Antifertility activity	Pathak et al. (2005)
	Roscoe				
	*Zingiberaceae*				
	*Sunthi*				
	Rhizome				
D Antiovulatory activity					
1.	*Achyranthes aspera* L.	Fatty acids, oleonic acid, bisdesmosidic, triterpenoid alkaloids, D-glucuronic, betaine, and achyranthine	Benzene, ethanolic, chloroform	Antiadulatory, anti-implantation, hormonal disturbance in uterus, and expulsion of ovary	Shibeshi et al. (2006); Vasudeva and Sharma (2006)
	Amaranthaceae				
	*Apamarga*Whole plant,				
	Stem bark, Root				
2.	*Areca catechu* L.	Alkaloids—pilocarpine, arecaidine, and arecoline	Ethanolic extract	Antiovulatory, ovarian weight decreased due to imbalance in gonadotrophins	Shrestha et al. (2010)
	Arecaceae				
	*Poogaphala,* Nuts				
3.	*Azadirachta indica* A. Juss.	Azadirachtin, nimbolinin, nimbin, nimbidin, nimbidol, sodium nimbinate, and gedunin	Alcoholic extract flower in Sprague–Dawley rats	Disrupted the estrous cycle and caused a partial block in ovulation	Gbotolorun et al. (2003); Vyas and Purohit (2018)
	Meliaceae				
	*Nimba*				
	Leaves, flower, and seed				
4.	*Butea monosperma* (Lam.) Kuntze	Kino-tannic acid, gallic acid, and pyrocatechin	Aqueous extract	Inhibit ovulation	Shrivastava et al. (2007), Sinha and Nathawat (1989)
	Fabaceae				
	Palash, bark, and flowers				
5.	*Calotropis procera* (Aiton) W.T. Aiton	Steroidal alkaloid	Calotropin, aqueous ethanol	Antiovulatory prolonged di-estrous stage with temporary inhibition of ovulation	Gupta et al. (1990); Abdelgader and Elsheikh (2018); Sharma and Jacob (2001a); Pokharkar et al. (2010)
	Apocynaceae				
	*Arka,* Root				
6.	*Catunaregam spinosa (Thunb.) Tirveng.*	Saponins, valeric acid resin, wax, and coloring matter	Ethanolic extract, isolated oleic acid	Antiovulatory effect in rabbits, antiimplantation activity in albino rats	Malhi and Trivedi (1972); Pillai et al. (1977)
	Rubiaceae				
	*Madanphal,*				
	Fruits, seeds, and pulp				
7.	*Citrus × aurantium* L	Citroflavanoids, glucosides, and triterpenoids	Petroleum ether	Anti-implantation, antiovulatory, abortifacient, increased ovarian weight, decreased Graafian follicles, irregular estrous cycle	Patil and Patil (2013)
	Rutaceae				
	*Bijaura,* Seeds				
8.	*Curcuma longa* L.	Curcumin and flavanoids	Ethanol, aqueous	Propylene glycol solution antifertility, antiovulatory, decreased ovarian weight, suppression of GnRH	Ghosh et al. (2011)
	Zingiberaceae				
	*Haldi,* rhizome				
9.	*Hibiscus rosa-sinensis* L.	Cyclopeptide alkaloid	Ethanol, benzene extract	Anti-implantation, antiovulatory, increased uterine weight, secretion of estrogenic by atretic follicles, postcoital antifertility	Neeru and Sharma (2008)
	Malvaceae				
	*Japa,* Flowers				
10.	*Musa paradisiaca* L.	Alkaloids and flavonoids	Ethanolic	Antiovulatory suppressed ovulation due to inhibition in secretion of GnRH	Soni et al. (2013)
	Musaceae*, Banana,* stem				
11.	*Papaver somniferum* L.	Noscapine alkaloid	Alcoholic extract	Antiovulatory decreased production of gonadotrophin	Kumar and Sachin (2013)
	Papaveraceae				
	*Ahiphen,* Latex				
12.	*Plumbago rosea* L.	Plumbagin, sitosterol glycoside, tannins, and fatty alcohol	Acetone, ethanolic	Antiovulatory inhibition of ovulation with irregular estrous cycle	Sheeja et al. (2011)
	Plumbaginaceae				
	*Raktachitrak,* Leaves				
13.	*Semecarpus anacardium* L.f. *Anacardiaceae*	Alkaloids	Aqueous and ethanolic	Reversible antiovulatory activity	Sushma et al. (2016)
	Bhallatak				
	Fruits				
14.	*Taxus baccata* L.	Pseudo alkaloids	Leaf extract	Antiovulatory, inhibited secretion of ovarian hormones	Priya et al. (2012); Kaur et al. (2011)
	Taxaceae				
	*Talishpatra Common Yew*				
	Leaves				
15.	*Vitex negundo* L.	Casticin, isoorientin, chrysophenol D, luteolin, p–hydroxybenzoic acid, and D-fructose	Aqueous	Antiovulatory activity	Lal et al. (1992)
	Lamiaceae				
	*Nirgundi,* roots, and seeds				
E Antiestrogenic activity					
1.	*Allium sativum* L.	Sulfur-containing compounds	Alcohol	Ecobolic in mice and rats, estrogenic activity in female albino rats	Tewari et al. (1971); Ola-Mudathir et al. (2008)
	Amaryllidaceae				
	*Rason,* Bulb				
2.	*Cyperus rotundus* L.	Cyperene, humulen, selinene, zierone, campholenicopaene, and limonene	Aqueous	Antiestrogenic property	Gediya et al. (2011)
	Cyperaceae				
	*Musta,* Rhizome				
3.	*Glycyrrhiza glabra* L.	Triterpene glycyrrhizin acid and glycoside	Water	Estrogenic activity	Ahmad et al. (2011)
	Fabaceae				
	*Yashtimadhu,* Roots				
4.	*Guilandina bonduc* L. *sy*. *Caesalpinia bonduc* (L.) *Roxb.*	Phytosterinin, β-sitosterol, flavonoids, bonducellin, aspartic acid, arginine, and citrulline β-carotene	Aqueous	Antiestrogenic activity	Salunke et al. (2011)
	Leguminosae				
	*Karanja,* seeds				
5.	*Nelumbo nucifera* Gaertn. Nelumbonaceae	Hydrocarbons	Ethanolic extract	Antiestrogenic, decreased ovarian weight, estrogens inhibition	Mutreja et al. (2008)
	*Kamala, Lotus*				
	Seeds				
6.	*Sesamum indicum* L.	Oil, protein, and carbohydrate	Extract	Estrogenic effect in female albino rats	Priya et al. (2012)
	Pedaliaceae				
	*Tila,* seeds				
7.	*Vitex negundo* L.	Casticin, isoorientin, chrysophenol D, luteolin, p–hydroxybenzoic acid, and D-fructose	Aqueous	Antiovulatory activity	Lal et al. (1992)
	Lamiaceae				
	*Nirgundi,* roots and seeds				
F Antispermatogenic activity					
1.	*Abru sprecatorius* L.	Abrin, abrasine, precasine, and precol	Aqueous	Reduced sperm motility, density, antispermatogenic effect, reduced activity of testicular enzyme, post-testicular antifertility effect	Bajaj et al. (1981); Dixit et al. (1987); Kulshreshtha and Mathur (1990); Sinha (1990)
	Papilionaceae				
	*Gunja,* seeds				
2.	*Aegle marmelos* (L.) Corrêa*.*	Marmelosin, luvangetin, psoralen, tannins, and marmin	Aqueous extract	Inhibit spermatogenesis and sperm motility male rat reproduction, affecting the sexual behavior and epididymal sperm concentration	Sur et al. (1999); Sur et al. (2002)
	Rutaceae				
	*Bilva,* whole plant and leaves				
3.	*Albizia lebbeck* (L.) Benth.	Melacacidin, D-catechin, β-sitosterol, albiziahexoside, betulnic acid, and echinocystic acid glycosides	Methanolic extract	Spermatogenic arrest in male albino rats	Gupta et al. (2004); (Gupta et al. 2005a)
	Fabaceae				
	*Shirish,* Pods				
4.	*Andrographis paniculata* (Burm.f.) Nees	Andrographolide, Andrographidoids A, B, C, D, E, diterpenoid, and lactone	Water extract	Antispermatogenic	Akbarsha et al. (1990); Akbarsha and Murugaian (2000)
	Acanthaceae				
	*Kirattikta,* leaves				
5.	*Ananas comosus* (L.) Merr.	Atropine alkaloids and anonaine	Water	Antispermatogenic activity	Satyawati (1983)
	Bromeliaceae				
	*Custard apple,* seeds				
6.	*Annona squamosa* L.	Atropine alkaloids and anonaine	Ethyl acetate extract	Antispermatogenic activity	Jain and Dixit (1992)
	Annonaceae				
	*Custard apple*				
	Seeds, leaves, and bark				
7.	*Areca catechu* L.	Alkaloids—pilocarpinearecaidine, arecoline	Water	No abnormality in Leydig cell and interstitium tissue	Ave Olivia et al. (2020)
	Arecaceae				
	*Poogaphala,* Nuts				
8.	*Aristolochia indica* L.	Aristolochic acid, ceryl alcohol, β-sitosterol, stigmast-4-en-3-one, friedelin, and cycloeucalenol	Aristolochic acid	Antispermatogenic	Gupta et al. (1996)
	Aristolochiaceae				
	*Ishwari,* roots				
9.	*Azadirachta indica* A. Juss.	Azadirachtin, nimbolinin, nimbin, nimbidin, nimbidol, sodium nimbinate, and gedunin	Aqueous, alcoholic	Decrease in the weight of seminal vesicles, ventral prostate, reduction in epithelial height, nuclear diameter, and the secretory materials in the lumen	Gediya et al. (2011)
	Meliaceae				
	*Nimba*				
	Leaves, flower, and seed				
10.	*Bacopa monnieri* (L.) Wettst.	Bacosides and saponins	Aqueous extract	Reversible suppression of spermatogenesis and fertility, without producing apparent toxic effects	Singh et al. (2013)
	Plantaginaceae				
	*Brahmi,* whole plant				
11.	*Balanites roxburghii*	Saponin, furanocoumarin, and flavonoid	Methanol, palmitine hydroxide	Antispermatogenic activity	Dixit et al. (1981), Agarwal and Dixit (1982)
	Planch.				
	Zygophyllaceae				
	*Ingudi,* Fruit pulp				
12.	*Berberis aristata* DC.	Berberine and berbamine	Palmitine hydroxide	Antispermatogenic action	Gupta and Dixit (1989)
	Berberidaceae				
	*Daruharidra,* Roots				
13.	*Butea monosperma* (Lam.) Kuntze	Kino-tannic acid, gallic acid, and pyrocatechin	Aqueous extract	Antispermatogenic effect	Wati and Verute (1988)
	Fabaceae				
	Palash, bark, and flowers				
14.	*Calotropis procera* (Aiton) W.T. Aiton	Steroidal alkaloid	Calotropin, aqueous ethanol	Antispermatogenic, antiandrogenic activities, and/or endocrine disrupting effects, functional alteration in genital organ	Gupta et al. (1990); Abdelgader and Elsheikh (2018); Sharma and Jacob (2001b) Pokharkar et al. (2010)
	Apocynaceae				
	*Arka,* root				
15.	*Carica papaya* L.	Papain, caricacin, carpasemine, oleanolic glycoside,	Pet ether, Alcohol, aqueous Ethanol	Antispermatogenic activity reduced spermatogenesis, inhibition in steroidal hormones	Changamma and Lakshman (2013)
	Caricaceae				
	*Papaya*, unripe fruit pulp,				
	seeds, latex				
16.	*Celastrus paniculatus*	Alkaloids, tannins, saponins, steroid, terpenoid, flavonoids, phlobatannin, cardiac, and glycoside	Seed	Antispermatogenic activity	Bidwai et al. (1990)
	Willd.				
	Celastraceae				
	*Jyotishmati*, seeds				
17.	*Cichorium intybus* L.	Inulin, sesquiterpene lactones, vitamins, minerals, fat, and mannitol,	Aqueous	Antispermatogenic activity	Roy and Venkatakrishna (1983)
	Asteraceae*, Chicory*				
	Whole plant				
18.	*Cinnamomum camphora* (L.) J.Presl	Essential oil—camphor, linalool, and cineole	Leaf	Inhibition of spermatogenesis	Singh (1990b)
	Lauraceae				
	*Karpur*				
	*Camphor,* leaves and resin				
19.	*Cuminum cyminum* L*.*	Cuminal and cuminic alcohol	Extract	Antispermatogenic effect	Priya et al. (2012); Sharma J et al. (2001)
	Apiaceae				
	*Jeerak,* seeds				
20.	*Embelia ribes* Burm.f.	Embelin, volatile oil, and fixed oil	Isolated embelin	Inhibition of spermatozoa motility	Prakash (1981); Nand (1981); Dixit et al. (1983); Gupta et al. (1989)
	Primulaceae				
	*Vidang,* berries				
21.	*Euphorbia neriifolia* L.	β-amyrin acetate, lupenone, 3-acetoxy-20-lupanol, cycloart-25-en-3β, 24ζ-diol, and cycloart	Ethanol	Antispermatogenic effect	Mali (1999)
	*Milk brush*				
	Euphorbiaceae				
	Latex, Whole plant				
22.	*Hibiscus rosa-sinensis* L.	Cyclopeptide alkaloid	Ethanol, benzene extract	Spermatogenic elements of testis and epididymal sperm count., androgenic activity	Reddy et al. (1997); Gupta et al. (1985)
	Malvaceae				
	*Japa*				
	Flowers				
23.	*Momordica charantia* L.	Glycosides, saponins, alkaloids, fixed oils, triterpenes, proteins, and steroids	Aqueous	Antispermatogenic, antisteroidogenic activity	Naseem et al. (1998)
	Cucurbitaceae				
	*Karwellaka*				
	Roots and leaves				
24.	*Ocimum sanctum* L.	Carvacrol, sesquiterpene, hydrocarbon, and caryophyllene	Benzene extract	Decreased sperm count, weight of testis, and sperm motility	Pandey and Madhuri (2010)
	Lamiaceae*, Tulsi,* leaves				
25.	*Piper betle* L*.*	Eugenol, eugenol acetate, piper betol, piperol, methyl eugenol, and phytol	Alcoholic extract	Reduced sperm motility	Adhikary et al. (1989); Sarkar et al. (2000)
	Piperaceae				
	*Betel leaf, Pan*				
	Petiole				
26.	*Piper nigrum* L.	Piperine	Fruit powder—suspended in sterile distilled water containing milk powder	Alterations in the male reproductive organs, reversible after cessation of treatment	Mishra and Singh (2009), Malini et al. (1999)
	Piperaceae				
	*Marich, Black pepper*				
	Fruit				
27.	*Plumbago zeylanica* L.	Plumbagin	Ethnol	Antispermatogenic	Purohit et al. (2008)
	Plumbaginaceae				
	*Chitrak,* Root				
28.	*Pterocarpus santalinus* L.f.	Santalin A, B, savinin, calocedrin, pterolinus K, L, and pterostilbenes	Water	Semen coagulating activity	Dhawan et al. (1980)
	Fabaceae				
	*Raktachandan*				
	Stem bark				
29.	*Pueraria tuberosa* (Willd.) DC.	Puerarin, genistein, and daidzein	Methanol	Inhibition of spermatogenesis	Gupta et al. (2004), Gupta et al. (2005b)
	Fabaceae*, Varahikand,* rhizome				
30.	*Semecarpus anacardium* L.f.	Bhilwanols, phenolic compounds, biflavonoids, and sterols glycosides	Ethanolic	Reduction in the number of primary spermatocytes, secondary spermatocytes, and spermatids	Gupta et al. (2013); Sharma et al. (2003)
	Anacardiaceae				
	*Bhallatak, Marking nut,* Seeds				
31.	*Terminalia arjuna* (Roxb. ex DC.) Wight &Arn.	Tannins, triterpenoid saponins, flavonoids, gallic acid, ellagic acid, and phytosterols	Crude form	Inhibition of spermatogenesis	Jha and Dixit (1986), Lal and Udupa (1993)
	Combretaceae				
	*Arjuna,* Bark				
32.	*Tylophora asthmatica* (L. f.) Wight &Arn*.* Apocynaceae	Aempferol, quercetin, tyloindane, cetyl-alcohol, tannins, glucose, calcium salts, and potassium chloride	Pure alkaloid	Antispermatogenic activity	Dikshith et al. (1990)
	*Khadki Rasna*				
	Leaf and stem				
G Spermicidal activity					
1.	*Acacia concinna* (Willd.) DC.	Hexacosanol, spinasterone, oxalic, tartaric, citric, succinic, ascorbic acid, alkaloids calyctomine, and nicotine	Alcoholic	Spermicidal and semen coagulating activity	Kamboj and Dhawan (1982)
	Leguminosae*-*Mimosoideae				
	*Shikekai,* stem bark				
2.	*Achyranthes aspera* L.	Fatty acids, oleonic acid, bisdesmosidic, triterpenoid alkaloids, D-glucuronic, betaine, and achyranthine	Benzene, ethanolic, and chloroform	Spermicidal	Raj et al. (2011); Shibeshi et al. (2006); Vasudeva and Sharma (2006)
	Amaranthaceae				
	*Apamarga*Whole plant,				
	Stem bark, Root				
3.	*Alstonia scholaris* (L.) R.Br. Apocynaceae	Erythrodiol, uvaol, betulin, oleanolic acid ursolic acid, and β-amyrin	Water extract	Decline germ cell population	Gupta et al. (2003), 2004)
	*Saptaparna,* stem bark				
4.	*Azadirachta indica* A. Juss.	Azadirachtin, nimbolinin, nimbin, nimbidin, nimbidol, sodium nimbinate, and gedunin	Aqueous and Alcoholic	Spermicidal effect on number of spermatozoa and level of fructose	Gediya et al. (2011), Kasturi et al. (1997)
	Meliaceae				
	*Nimba*				
	Leaves, flower, and seed				
5.	*Bambusa bambos* (L.) Voss	Balarenone, barlerin, barlerinosideverbascoside, acetylbarlerin, and lupulinoside	Ethanolic	Reduced sperm motility	Vanithakumar et al. (1989)
	Poaceae*, Vansha*				
	Tender stem				
6.	*Cannabis sativa* L.	Cannabinoids, terpenes, and sesquiterpenes	Butin	Testicular lesions	Dixit and Joshi (1982)
	Cannabaceae				
	*Bhanga,* leaves				
7.	*Citrullus colocynthis* (L.) Schrad.	Carbohydrate, protein, amino acid, tannins, saponins, phenolics, and cardicglycoloids	Ethanol	Impairment of sperm	Chaturvedi and Dixit (1997)
	Cucurbitaceae				
	*Indrawaruni*				
	*Bitter apple,* fruits				
8.	*Daucus carota* L.	Essential oil	Petroleum, ether, benzene, alcohol, and water	Spermicidal activity	Garg (1975); Jansen and Wolhlmuth (2014); Shah and Varute (1980)
	Apiaceae				
	*Grinjanak,* Seed				
9.	*Embelia ribes* Burm.f.	Embelin	Embelin in 50 and 100°mg/kg doses	Reversible contraception like activity in male dogs	Nand (1981); Dixit and Bhagava (1983)
	Primulaceae				
	*Vidang,* Berries				
10.	*Mentha arevensis* L.	Alkaloids, steroids, and glycosides	Petroleum ether	Spermicidal Decreased weight of testis, sperm motility, and viability	Sharma and Jacob (2001a)
	Lamiaceae				
	*Pudina,* leaves				
11.	*Myristica fragrans* Houtt	Myristicin, elemicin, myristic acid, alpha-pinene, terpenes, beta-pinene, and trimyristin	Ethanol	Premature ejaculation	Mishra and Shukla (1980)
	Myristicaceae				
	*Nutmeg, Jatiphal,* seeds				
12.	*Strychnos potatorum* L.f.	Strychnine	Seed extract	suppressive effects on male fertility	Gupta et al. (2006)
	Loganiaceae				
	*Nirmali,* Seeds				
13.	*Terminalia bellirica* (Gaertn.) Roxb.	Phenolic acids, saponins, lignans, triterpenoids, resveratrol glycosides, arjungenin, β-sitosterol, and stigmasterol	Aqueous	Spermicidal activity in rat semen, human semen	Kaur et al. (2011)
	Combretaceae				
	*Bibhitak*				
	Fruits				
14.	*Tinospora cordifolia (*Willd.) Hook.f.	Berberine, palmatine D, choline D, diterpene, terpenoids alkaloids, and steroids	Aqueous	Spermicidal Reduced weight of testis, sperm count	Gupta and Sharma (2003)
	& Thomson				
	Menispermaceae				
	*Amrita Giloe*				
	Stem				
15.	*Trigonella foenum-graecum* L., Fabaceae	Water, carbohydrates, protein, fat, and calcium	Aqueous	Spermicidal activity in human and rat semen	Priya et al. (2012)
	*Methika,* Seeds				
16.	*Withania somnifera* (L.) Dunal	Withanolides	Stem, ethanolic	Reversible spermicidal and infertilizing effect	Singh et al. (2013); Mali (1999)
	Solanaceae				
	*Ashwagandha*				
	Stem and root				
H Antiandrogenic activity					
1.	*Aloe barbadensis* Mill.	Water, polysaccharides, pectin, cellulose, hemicellulose, and glucomannan	Extract	Antiandrogenic activity on monkeys	Dixit et al. (1983)
	Synonym of Aloe vera (L.) Burm.f.				
	Asphodelaceae				
	*Kumari,* leaves				
2.	*Aristolochia indica* L.	Aristolochic acid, ceryl alcohol, β-sitosterol, stigmast-4-en-3-one, friedelin, and cycloeucalenol	Aristolochic acid	Antiandrogenic effects on langur monkey	Gupta et al. (1996)
	Aristolochiaceae				
	*Ishwari,* roots				
3.	*Andrographis paniculata* (Burm.f.) Nees	Andrographolide, andrographidoids A, B, C, D, E, diterpenoid, and lactone	Water extract	Antiandrogenic	Akbarsha et al. (1990); Akbarsha and Murugaian (2000)
	Acanthaceae				
	*Kirattikta,* leaves				
4.	*Azadirachta indica* A. Juss., Meliaceae	Azadirachtin, nimbolinin, nimbin, nimbidin, nimbidol, sodium nimbinate, and gedunin	Seed oil	Antiandrogenic	Sharma et al. (1987); Sinha et al. (1984); Roop et al. (2005)
	*Nimba*				
	Leaves, flower, and seed				
5.	*Cuscuta reflexa* Roxb	Alkaloids	Methanolic	Antisteroidogenic	Gupta et al. (2003)
	Convolvulaceae				
	*Amarwel,* whole plants				
6.	*Curcuma longa* L.	Curcumin and flavanoids	Ethanol, aqueous	Antiandrogenic	Bhagat and Purohit (1986)
	Zingiberaceae				
	*Haldi,* rhizome				
7.	*Foeniculum vulgare* Mill	Anethole, alpha pinene, beta myrcene—pinene, bitter fenchone, camphene, and estragole	Alcoholic	Antiandrogenic	Farooq et al. (1997)
	*Apiaceae*				
	*Common fennel, seeds*				
8.	*Hibiscus rosa-sinensis* L.	Cyclopeptide alkaloid	Ethanol and Benzene extract	Spermatogenic elements of testis and epididymal sperm count., androgenic activity	Reddy et al. (1997); Gupta et al. (1985)
	Malvaceae				
	*Japa, Flowers*				
9.	*Mucuna urens* (L.) Medik.	L-DOPA, with trace amounts of serotonin, nicotine, and bufotenine	Water	Effect on gonads and sex accessory glands	Udoh and Ekpenyong (2001)
	Fabaceae				
	*Horase been, Kapikacchu*				
	Seeds				
10.	*Nicotiana tabacum* L*.*	Lipid constituents, free fatty acids, triglycerides, and sterol esters free sterols	Nicotine	Antiandrogenic	Londonkar et al. (1998)
	Solanaceae				
	*Tobacco,* leaves				
11.	*Plumbago zeylanica* L.	Plumbagin	Plumbagin-free alcohol	Antiandrogenic	Bhargava (1984)
	*Plumbaginaceae*				
	*Chitrak,* root				
12.	*Ruta graveolens* L.	Volatile oil	Aqueous extracts	Adverse effects on territorial aggression and sexual behavior in male albino rats	Khouri and Akawi (2005)
	Rutaceae*, Rue,* leaves				
13.	*Semecarpus anacardium* L.f.	Bhilwanols, phenolic compounds, biflavonoids, and sterols glycosides	Aqueous extracts	Antiandrogenic	Singh (1985)
	Anacardiaceae				
	*Bhallatak, Marking nut,* Seeds				

Pharmacologically, there are about 67 medicinal plants which possess antifertility activity in females and 56 medicinal plants in males. Several plants have shown to help contraception from the female and male perspectives.

In various experimental animal models, these herbal extracts have shown minimal side effects in comparison to the chemically synthesized contraceptives, which usually contain various combinations of hormones. These plant extracts have active phytoconstituents, which are responsible for the antifertility effects such as antiovulation, anti-implantation, and others.

## Clinical Studies

Some of the plants that have demonstrated interesting antifertility activity in clinical trials are as follows.

### *Embelia ribes* Burm.f.

Single drug was administered in a dose of 2°g for 5°days followed by 1°g daily for another 10°days. After observing the effect on 2051 cycles in 45 women over 4°years, it was reported that the plant protected 95% of women from pregnancy (Tewari et al., 1976).

### *Hibiscus rosa-sinensis* L.

Red petals of the plant *Rudrapushpaka* collected between October and December. The extract was administered to 30 sexually active women at a dose of 750°mg/day from day 7 to day 22 of the reproductive cycle. It was observed that no one had become pregnant (Tewari, l974).

### Neem oil

A study was conducted on neem seed oil as local application for the reproductive female [246 women in the fertile age-group, 4 dropped out] as a method of family planning for a period of 12–36 cycles. In nine cases, there was conception due to drug failure and in four cases, there was conception due to drug omission. Neem seed oil may be used as an external barrier as a cost-effective herbal contraceptive for its spermicidal property and is considered safe for regular use. (Achintya, 2018).

### *Ricinus communis* L.

The seeds of *Ricinus communis Linn* RICOM-1013-J, administered as a single oral dose of 2.3–2.5°g once/12°months acted as protection against pregnancy in 50 women volunteers. The study revealed very minimal side effects. The antifertility and contraceptive efficacy of RICOM-1013-J is due to hormonal mechanisms (Isichei et al., 2000). Goncim et al. (2010) stated that one seed of *Ricinus communis L*. taken orally can prevent ovulation in humans and the anticonceptive effect may be due in part to the prevention of ovulation.

### Compound Formulation

A study was conducted on a combination of *Ashoka (Saraca indica* L*.), Vidanga (Embelia ribes Burm.f* ***.*** *), Laksha (lac),* and *Kramuk (Areca nut)* on 834 young, healthy patients in active reproductive age below 40°years. The drug was administered from the 5th°day of LMP for a period of 15°days in a daily dose schedule of 1°gm (2 tablets) at bedtime with milk. Results suggested that the failure rate of treatment 1.19/HWY is comparable to both steroidal oral contraceptive pills and intrauterine device. It does not affect the hypothalamo-pituitary axis and did not have any other adverse effects. It can be a good alternative for lactating women (Palep and Jukar, 2003).

Central Council for Research in Ayurveda and Siddha had taken up a number of studies to evaluate the efficacy of Ayurvedic formulations like *K Capsule, Ayush AC-IV, Pippalyadi yoga (in three different doses), Ayush AC II, Talisadi yoga, Vidangadi yoga*, etc., which were proved as safe and effective in different clinical studies. Besides this, the council also tried the efficacy of *neem oil*—as a local contraceptive and found encouraging results (Galib et al., 2008).

## Teratogenic Effect

Ayurveda classical texts have references to congenital birth [*anmabalapravrita*] disorders as per the etiopathology and clinical presentation. Some congenital malformations in the fetus may occur but the mechanism is still not clear.

Teratogen is an agent or factor that causes malformation in the embryo. One of the causes of malformation may be toxic substances such as drugs and environmental toxins in pregnancy.

Herbal drugs with appropriate dose and duration may not cause teratogenic effect but in the case of excess dose with improper mode of administration, for a longer duration than therapeutically advised, teratogenic effect may be seen. Scientific validation of their safe use in pregnancy is hardly documented. Teratogenic effects of some of the medicinal plans have been mentioned in Table 3.

**TABLE 3:** List of drugs with teratogenic effect

Sr. No.	Name of plants	Phytoconstituent	Dose and duration	Teratogenic effect
1	*Asparagus racemosus* Willd.	Shatavarin, Racemosol	1000°mg/kg/body weight for 60-day Charles foster rat pups	Prenatal study—increased resorption of fetus, gross malformation i.e., swelling in legs, IUGR with small placental size.
	Root		Methanolic extract	Postnatal study—decreased number of pups per litter and increased mortality of pups and delayed developmental parameters Goel et al. (2006)
2	*Datura metel* L.	Atropine alkaloids	500°mg/body kg wt rats, ethanolic extract	Teratogenic in the late stage of pregnancy Azeez and Philip (2013)
	Leaves			
3	*Gloriosa superba* L.	Colchicine	1-3 ppm and 4-5 ppm	Antifertility activity scarcely produced abnormal embryos. Induce high percentage of abnormalities. Badwaik (2011)
	Tuber		Hydroalcoholic extract	
4	*Lawsonia inermis* L.	Flavonoid and phenolic compounds	100°mg/kg body wt. BALB/c mice between 8-12°wk hydroalcoholic extract	90% embryo, more extra ribs anencephaly, exencephaly, skeletal abnormalities, height and weight loss in embryos Lobat (2015)
5	*Luffa operculata* (L.) Cogn.	Glycosides, saponins, resin, free sterols, aliphatic esters, quinones	After ingestion of a variable amount of tea made with dried fruit, decoction	Abortion,reduction in birth rate Barilli et al. (2005)
	Tea, decoction			
6	*Plumbago zeylanica* L*.*	Plumbagin	100°mg/body kg wt orally with 0.5°ml of distilled water in mice	Stunted growth, subcutaneous, and deep hemorrhage, kinking of tail, protrusion of back of head Srivastava (2017)
7	*Ruta graveolens* L.	Essential oil	5, 10, and 20% w/v or plain water (control) orally for 4 days	Changes in the blastocyst formation, reducing the number, and delaying the development of embryos Gutiérrez-Pajares et al. (2003) embryotoxic effect De Freitas et al. (2005)
8	*Sena (Senna) alexandrina Mill-*Fabaceae	Sennosides	Extract	Increase blood flow to the uterus and its attachments, increasing the risk of fetal loss, and may pass spasms in the infant
				Schulz et al. (2002)
9	*Zingiber officinale*	Carbohydrates (50–70%), lipids (3–8%), terpenes, and phenolic compounds	Orally at 0, 250, 500, 1000, or 2000 °mg/kgbw/day—five groups	High dose significantly reduced the number of live fetuses, increased fatal death, and resorption. Reda et al. (2018)
	Roscoe			
10	*Pipalyadi gutika*	Piperine	5 times to one and five times to the other than the recommended dose for humans Rats	Fetus—LBW, smaller in length, developmental defects of soft tissues, skeletons, herniation of intestines into umbilical cord,
				Mother—less weight gain during gestation Chaudhury et al. (2001)
11	*Vishamustivati [VV] & Shuddha Tankana [ST]*	-	175°mg/kg of aqueous solutions of VisamustiVati, 300°mg/kg aqueous solutions of SuddhaTankana, orally from day 1 to day 7 of post mating period	VV and ST shows positive Teratological effect on new-borns, gross remarkable external morphological and skeletal defects Jati (2018)

It is observed that drugs having contraceptive and abortifacient action have potent teratogenic effect in experimental models. There are several studies of teratogenicity on other herbal drugs which are not showing teratogenic effects in low doses and may cause teratogenic effects in high doses, for example, *Ashwagandha (Withania somnifera* (L.) Dunal*), Punarnava (Boerhavia diffusa* L*.), Narangi (Citrus aurantium L), Nimba (Azadirachta indica* A. Juss.*), Jatamansi (Nardostachys jatamansi (D.Don) DC.), (Bala Abutilon indicum* L.) Sweet*),* and *Yastimadhu (Glycyrrhiza glabra* L.) (Jati, 2018).

Different contraceptive activities in the abovementioned 94 plant ingredients are categorized in Table 4.

**TABLE 4:** List of medicinal plants with one or more contraceptive activities.

Sr. No	Plant name	*Anti-implantation*	*Abortification*	*Antifertility*	*Antiovulatory*	*Antiestrogenic activity*	*Antispermatogenic*	*Spermicidal*	*Antiandrogenic activity*
1	*Abies spectabilis* (D.Don) Mirb.	√							
2	*Abroma augusta (L.) L.f.*	√	√						
3	*Abrus precatorius* L.		√	√			√		
4	*Acacia concinna (Willd.) DC.*							√	
5	*Acacia leucophloea (Roxb.) Willd.*			√					
6	*Achyranthes* aspera L.		√		√			√	
7	*Adhatoda vasica* Nees	√	√						
8	*Aegle marmelos (L.) Corrêa.*		√				√	√	
9	*Ailanthus excelsa Roxb*	√							
10	*Albizia lebbeck (L.) Benth.*						√		
11	*Allium cepa L.*	√							
12	*Allium sativum* L.					√			
13	*Aloe barbadensis Mill.*	√							√
	*Synonym of Aloe vera (L.) Burm.f.*								
14	*Alstonia scholaris* (L.) R.Br.						√		√
15	*Andrographis paniculata (Burm.f.) Nees*						√		√
16	*Ananas comosus* (L.) Mer							√	
17	*Annona squamosa* L.		√				√		
18	*Areca catechu* L.	√	√	√	√		√		
19	*Aristolochia indica* L.						√	√	
20	*Azadirachta indica A. Juss.*			√	√		√	√	√
21	*Bacopa monnieri (L.) Wettst.*						√		
22	*Balanites roxburghii Planch.*						√		
23	*Bambusa bambos* **(L.) Voss**							√	
24	*Barleria prionitis* L.		√						
25	*Berberis aristata* DC						√		
26	*Butea monosperma (Lam.) Kuntze*				√		√		
27	*Calotropis procera* (Aiton) Dryand.				√		√		
28	*Cannabis sativa* L.							√	
29	*Carica papaya L.*	√	√	√			√		
30	*Cassia fistula* L.	√							
31	*Catunaregam spinosa (Thunb.) Tirveng.*								
32	*Celastrus paniculatus Willd.*						√		
33	*Centratherum anthelminticum (L.) Gamble*	√							
34	*Cichorium intybus L.*						√		
35	*Cinnamomum camphora* (L.) J. Presl						√		
36	*Cissampelos* pareira L.			√					
37	*Citrullus colocynthis* (L.) Schrad.							√	
38	*Citrus × aurantium* L	√	√		√				
39	*Crateva nurvala* Buch. -Ham			√					
40	*Cuminum cyminum L.*			√			√		
41	*Cuscuta reflexa* Roxb								√
42	*Curcuma longa* L.			√					√
43	*Cyperus rotundus L.*					√			
44	*Daucus carota* L		√	√				√	
45	*Desmodium gangeticum* (L.) DC.			√					
46	*Embelia ribes* Burm.f.	√	√				√	√	
47	*Euphorbia neriifolia* L.						√		
48	*Ferula jaeschkeana* Vatke			√					
49	*Foeniculum vulgare* Mill								√
50	*Gloriosa superba* L.	√	√	√					
51	*Glycyrrhiza glabra* L					√			
52	*Grewia asiatica* L	√	√						
53	*Guilandina bonduc L*.					√			
	Sy. *Caesalpinia bonducella* (L.) Fleming								
54	*Hibiscus rosa-sinensis* L.	√		√	√		√		√
55	*Lawsonia inermis* L.			√					
56	*Lepidium sativum* L		√	√					
57	*Melia azedarach* L			√					
58	*Mentha arevensis* L							√	
59	*Mesua ferrea* L.	√							
60	*Michelia champaca* L.	√							
61	*Momordica charantia* L.	√		√			√		
62	*Mucuna urens* (L.) Medik								√
63	*Musa paradisiaca* L.				√				
64	*Myristica fragrans* Houtt							√	
65	*Nelumbo nucifera* Gaertn.					√			
66	*Nicotiana tabacum* L*.*								√
67	*Nigella sativa* L.			√					
68	*Ocimum sanctum* L.						√		
69	*Papaver somniferum* L.				√				
70	*Piper betle* L.			√			√		
71	*Piper longum* L.			√			√		
72	*Piper nigrum* L.			√			√		
73	*Plumbago rosea L.*				√				
74	*Plumbago zeylanica* L.	√					√		√
75	*Pterocarpus santalinus* L.f.						√		
76	*Pueraria tuberosa* (Willd.) *DC*						√		
77	*Ricinus communis* L.	√	√						
78	*Ruta graveolens* L								√
79	*Sapindus trifoliatus* L.	√							
80	*Semecarpus anacardium* L.f.				√		√		√
81	*Sesbania sesban* (L.) Merr	√							
82	*Sesamum indicum* L					√			
83	*Strychnospotatorum* L.f.							√	
84	*Taxus baccata* L				√				
85	*Terminalia arjuna* (Roxb. ex DC.) Wight &Arn						√		
86	*Terminalia bellirica (Gaertn.) Roxb*							√	
87	*Tinospora cordifolia* (Willd.) Hook.f.& Thomson							√	
88	*Trichosanthes cucumerina* L.			√					
89	*Trigonella foenum-graecum* L.							√	
90	*Tylophora asthmatica* (L. f.) Wight & Arn						√		
91	*Vitex negundo* L.				√	√			
92	*Withania somnifera* (L.) Dunal							√	
93	*Woodfordia fruticosa (L.)* Kurz		√						
94	*Zingiber officinale Roscoe*			√					

## Discussion

Presently, scientifically established methods of contraception and contraceptive drugs are used extensively. The synthetic contraceptive drugs known to interfere with the endocrine system and natural hormones may produce reproductive, neurological, developmental, and metabolic adverse effects that are serious at times. Search for safer drugs and preference for natural origin contraceptive drugs and methods are of research interests. Necessarily, the objectives for research of novel contraceptives from nature would be the assurance regarding effectiveness, safety, and user compliance. There are many plants known to have antifertility activity both in male and female. Some of these plants had spermicidal and altered hormone levels.

The classical Ayurvedic texts offer substantial knowledge on reproductive biology for healthy progeny and medieval Ayurvedic and specific Sanskrit texts provide information about methods and a broad range of therapeutics and ingredients that are described for use in contraception. These include local and oral contraceptives, abortifacients, and other methods of antifertility and birth control. These formulations and ingredients are a valuable source for extended research in the field of contraception.

In this study, 94 indigenous medicinal plants have been reviewed. Chemotaxonomically, it is of interest to note that the maximal number of plants having abortifacient and contraceptives are from *Fabaceae, Acanthaceae, Euphorbiaceae*, and *Liliaceae* families.

### Ingredients, Phytoconstituents, and Contraceptive Activities

Certain alkaloids, glycosides, saponins, tannins, terpenoids, and other phytoconstituents are known to disrupt ovarian functions and estrous cyclicity through interplay of ovarian and extra ovarian hormones. Alkaloids are a major group of secondary metabolites bitter in taste that stimulate the central nervous system or directly work on the human brain. These are antiparasitic, antiplasmodial, anticorrosive, antioxidative, antibacterial, anti-HIV, and have insecticidal activities. In a review, it has been suggested that maximum alkaloids containing plant drugs have been reported to have an antifertility, antiovulatory, anti-implantation, abortifacient effect on animals (Choudhury and Jadhav, 2013).

A majority of these medicinal ingredients used either in formulations or singly over centuries have also been studied for a variety of pharmacological, biological, and therapeutic activities.

#### *Achyranthes aspera* L.

A plant known to have antimicrobial, hypolipidemic, and has antifertility qualities is also used to treat asthma and cough.

#### Fruits of *Annona squamosa* L.

A known insecticidal, antiovulatory, and abortifacient plant that is hematinic, cooling, a sedative, stimulant, expectorant, and tonic. Its seeds are abortifacient and insecticidal and are used to destroy lice in the hair.

#### *Calotropis gigantea* L.

*Calotropis gigantea* L. having certain antifertility glycosides and cardenolides is used for colic pain, flatulence, asthma, cough, and whooping cough and has wound healing, anticancer, and hypoglycaemic effects. *Calotropis Madar* rootbark is used for abortive purposes and in India is used as an antidote and in the treatment of elephantiasis, leprosy, and chronic eczema.

#### Camphor

*Camphor*, the well-known aromatic, has hormone-modulating, contraceptive, abortifacient, and lactation-inhibiting properties in women. It has a dose-dependent effect in human sperm motility and viability. Camphor can pass the placental barrier and affect embryo development. Camphor-containing compounds have shown uterotrophicantitussive, anticonvulsant, nicotinic receptor blocking, anti-implantation, antiestrogenic, as well as estrogenic activities and can reduce serum triglyceride and thyroid hormone.

Flowers of *Hibiscus rosa-sinensis* L containing quercetin-7-O-galactoside, polyphenolic compounds, and kaempferol, having antispermatogenic compounds, is prescribed for contraception and is used to treat bacterial infection, hyperlipidemia, and depression and act as an antioxidant.

Two of the most bitter stimulant plants, *Momordica charantia* L. and *Azadirachta indica* A. Juss.*,* produce an irregular pattern of estrous cycle with prolonged diestrus phase. Steroids, triterpenoids, reducing sugars, alkaloids, phenolic compounds, flavonoids, and tannins in the plant cause reduction in the number of normal follicles because of atresia which occur due to disruption of the process of follicle selection*. Azadirachta* arrests spermatogenesis and androgen depletion.

Roots of *Plumbago zeylanicum* L. have been used as an abortifacient, internally or as an irritant to the uterus. This acrid and stimulant root increases appetite helps indigestion and is used for dyspepsia, piles, and skin diseases. It induces sweating, its powder is occasionally taken as snuff to relieve headache, and it helps in the adhesion of tissues in the body and is antidiarrheal.

*Tinospora cordifolia* (Willd.) Hook.f. and Thomson, an immunomodulator plant used to treat tuberculosis, fever, and wounds, has antifertility qualities. It is used for antioxidant, hypoglycaemic, and cardioprotective activities.

Excessive use of substances having pungent, bitter, and astringent tastes is contraindicated for sexual functions. Excess consumption of bitter taste leads to loss of strength and energy, astringent taste affects the sperm count, and can even reduce the sex drive while strongly pungent ingredients like pepper exhibit spermicidal or abortifacients effects. Prolonged consumption of these tastes may lead to emaciation of the body.

### Mechanism of Action

#### Female Contraceptives

Medicinal plants may induce infertility in distinct ways. They may affect the ovarian, uterine, and hormone production functions and interfere with implantation or sperm production. These drugs are of natural origin, hydrophilic, and lipophilic; can traverse paracellularly through the vaginal mucosa; and exhibit its efficacy as contraceptive, by altering the vaginal pH. These drugs may variably act locally to bring changes in the cervical mucus and alter decidual embedding and thereby act as anti-implantation agents, or may inhibit propulsion of sperm in the fallopian tubes by altering tubal mechanism or may act on hormones as antiovulation agents. They may act through rapid expulsion of the fertilized ova from the fallopian tube or inhibit implantation due to disturbance of the estrogen progesterone balance or induce fetal abortion by inhibition of nutrition to the uterus and the embryo.

Moreover, plants with estrogenic property can directly influence pituitary action by peripheral modulation of luteinizing hormone (LH) and follicle stimulating hormone (FSH), decreasing their secretions and blocking ovulation (Brinker, 1997). Plants with antiestrogenic activities intercept in the process of development of ovum and endometrium and on the other hand, plants have abortifacient effects (Gark et al., 1978; Prakash et al., 1985).

The site of action of antifertility agents in females comprises the hypothalamus, the anterior pituitary, the ovary, the oviduct, the uterus, and the vagina. The mammalian uterus is the main site of antifertility effects (Williamson et al., 1996). Typical estrogenic compounds posses the ability to increase the uterine wet weight and induce cornification and opening of vagina in immature rats, which results in anti-implantation effects (Turner, 1971).

Antifertility plants prevent fertilization; these drugs obstruct the formation of gametes and interfere with the process of fertilization. Antiovulatory plants induce infertility by suppressing ovulation. *Anti-implantation* plants prevent the attachment or penetration of fertilized ovum into the uterus. *Butea monosperma* (Lam.) Kuntze*, Ocimum sanctum* L., *Calotropis procera* (Aiton) W.T. Aiton, *Mentha arvensis*, and *Lawsonia inermis L*—all have anti-implantation activity. Abortifacient plants cause early expulsion of the fetus. These act during the first five weeks of pregnancy as they block the action of progesterone so that the uterus sloughs off the embryo. *Abrus precatorius* L., *Annona squamosa* L.*, Calotropis procera* (Aiton) W.T. Aiton, *Carica papaya* L.*, Dhatura metel* L*., Momordica charantia* L.*,* and *Catunaregam spinosa* (Thunb.) Tirveng are medicinal plant drugs which can be used as abortifacients. Stimulant, irritant, and bulk forming characteristics of these drugs facilitate abortion along with hormonal regulation and modulation of genital functioning. These ingredients are considered stimulants and are hot in nature and hence should be used for a short duration.

It observed that large numbers of antifertility plant extracts are known to exhibit estrogenic activity in rats (Dahanukar et al., 2000). Estrogenic substance may cause the expulsion of ova from the tube, disruption of luteotrophic activity of the blastocyst, and disrupt the functional equilibrium between the endogenous estrogen and progesterone, which may result in failure in fertility. Increase in the wet weight of uterus of substance-treated ovariectomized immature rats may indicate that the substance has an estrogenic effect (Mukherjee, 2002).

The hypothalamus has threshold requirement for estrogen to cause a massive release of LH by the pituitary gland. This surge of LH is the trigger, which initiates the rupture of the follicle (ovulation) (Bullock et al., 1995). It is known that an increase in the serum progesterone level prevents pregnancy through inhibition of ovulation and alteration of cervical mucus.

Most of the plants possess inhibition of implantation or reduction of estrogen level and increment of progesterone level as the possible mechanism of antifertility effect.

The anti-implantation effect may be due to the disturbance of endocrine–endometrial synchrony that is dependent on estrogen and progesterone balance. Factors other than the hormones such as histamine, prostaglandins, proteolytic enzyme NOS, alkaline phosphatase, interleukins, and leukemia-inhibitory factors, which are important for implantation, may also be affected by the various plant extracts (Gupta, 1994; Garg et al., 1978; Novaro et al., 1997; Prakash et al., 1989; Dimitriadis et al., 2003; Yang et al., 1994)

### Male Contraceptives

Male contraceptive drugs may inhibit spermatogenesis or act on male hormones when used orally or may be spermistatic or spermicidal when used intravaginally. Male contraceptives might work to suppress sperm production by antispermatogenic or prevent maturation of sperm or prevent the flow of sperm through the vas deferens or deposition of the sperm (Soni et al., 2015).

Plant extracts have also shown promising antifertility effects when administered to male rats. The various effects on male reproductive system to induce antifertility action shown by plants includes antispermatogenic effect, post-testicular antifertility effect, spermicidal effect, sperm-immobilizing effect, antiandrogenic effect, etc.

Antispermatogenic activity indicates interference in the steroidogenesis when the cholesterol level rises and sudanophilic lipid accumulates (Mandal et al., 2010). Some of the plant extracts kill the viability and work on Sertoli cells and have various effects on spermatogenesis, such as reducing the nuclear and cytoplasmic volume and vacuolizing Sertoli cells (Sharma RS et al., 2001) or acts through Leydig cells (Dufau et al., 1984). Some plant extracts act by unbalancing the hormones or through their antimotility activity (Verma and Yadav, 2021).

Spermicidals are contraceptive substances that destroy the sperm when inserted vaginally prior to intercourse. The spermicidal agents consist of a surfactant that destroys the sperm cell membrane. Lipid peroxidation may play an important role in disrupting the sperm membrane physiology that may or may not be accompanied with a detrimental effect on the defense system of the human spermatozoa against the ROS.

Antiandrogens, also known as androgen antagonists or testosterone blockers, prevent androgens like testosterone and dihydrotestosterone (DHT) from mediating their biological effects in the body. *Andrographis paniculata* (Burm.f.) Nees*, Azadirachta indica* A. Juss., *Curcuma longa* L., *Hibiscus rosa-sinensis* L*.,* and *Plumbago zeylanica* L. act by blocking the androgen receptor (AR) and/or inhibiting or suppressing androgen production. They can be considered as the functional opposites of AR agonists, for instance, androgens and anabolic steroids (AAS) like testosterone, DHT, and nandrolone and selective androgen receptor modulators (SARMs) like enobosarm.

**FIGURE 4:**
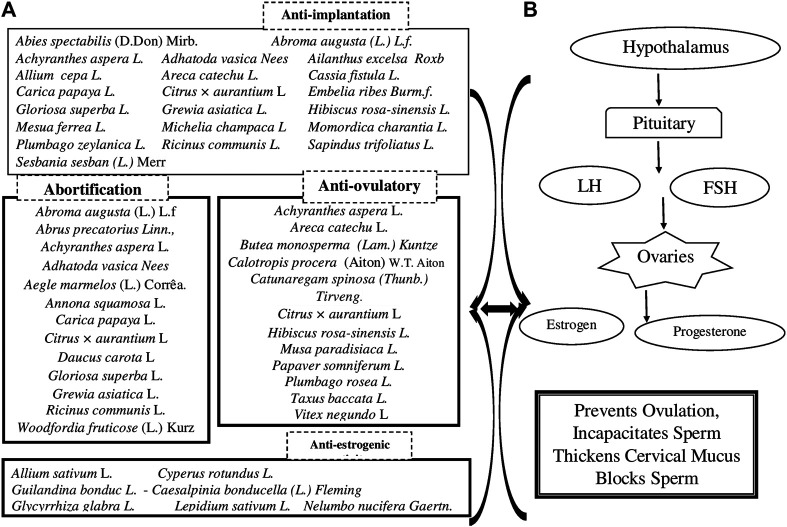
Female contraceptive plants and possible mechanism.

**FIGURE 5:**
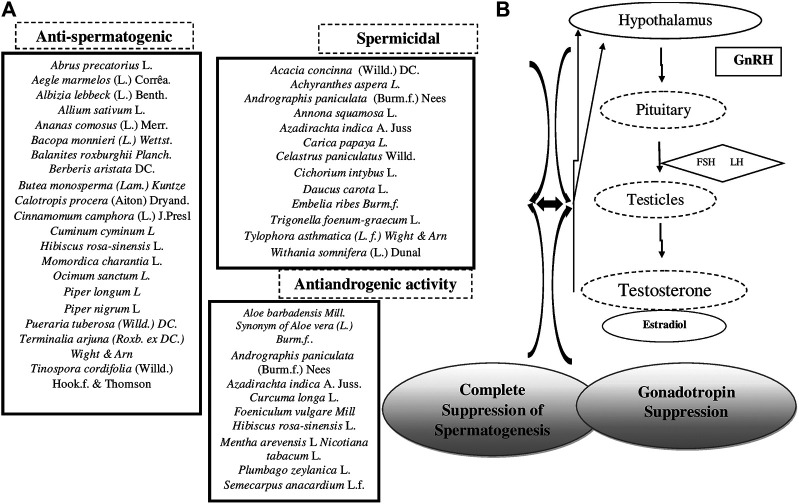
Male contraceptive plants and possible mechanism.

Figures 4, 5 provide group of these plants 3 (a) and 4 (a) with probable female and male contraceptive activities 3 (b) and 4 (b), respectively.

### Limitations/Challenges

A major limitation is the contradictory reports or non-reproducibility of published data, which can provide useful leads. At times, failure of reproducibility of contraceptive activity of a plant or its constituent is observed. This could be due to the multiple factors at different levels that are known to affect the reproductive process. The other reason could be the variable effect of the herbal contraceptive/s in animals as against when used in humans.

The contraceptives of natural origin are not used much in practice, the main factor being the lack of standardization and reliable validation studies. The information has thus remained fragmented. Studies have consequently been scarce. Interest has weaned due to the complexity and enormity of the large and long-term study requirements covering multiple variables.

Analytical methods, information on phytoconstituents, availability of markers, and their activities have now provided new standardization approaches to herbal products that assure higher safety and stability.

The solution to this is to investigate the efficacy of these herbs in humans themselves, after ascertaining their safety in animal models. There is also a need to record the conditions under which the plants are used by indigenous people, including the time and place of collection, proper botanical authentication, and schedule of administration. Advances in biology offer adaptable and promising experimental models to examine the effectiveness of natural products for altering reproductive functions and contraception

## Contraception and New Technologies for Natural Products

There is a need to use new contraceptive methods to minimize the side effects. The following technological advances are relevant in the context of this review for discovery and development of novel contraceptives of natural origin.

o Ayurveda recommends fumigation as a method and as a therapeutic procedure to treat various diseases, including microbial infections. Ayurvedic methods of sterilization with fumigation can be alternated as a modern contraceptive with the help of nanotechnology. Natural novel bioactive compound drugs could be developed with novel drug-delivery systems.
o A team in the University of Washington has developed an electrically spun cloth with nanometer-sized fibers that get dissolved to release drugs, thus providing a platform for cheap, discrete, and reversible protection ("Drug-Eluting Fibers for HIV-1 Inhibition and Contraception").
o Pharmacy on a chip is one of the most exciting parts of the drug-delivery system. It is a chip implanted into the body which releases drugs at set intervals. It would release the hormones estrogen and progestogen over a specific period to stop the release of eggs from the ovaries and thus prevent pregnancy.
o Nanotechnology-based condom systems have the potential to prevent the spread of HIV and STIs.
o Transdermal drug delivery (TDD) is an alternative method of drug administration for drugs whose delivery by conventional oral, topical, intravenous, and intramuscular methods is of limited efficacy. Recent advances in TDD involve the use of nanoparticles (NPs), which exhibit great potential to enhance drug permeation across the skin.
o Skin patches containing microneedles is a painless and minimally invasive method of TDD in which micron-sized pores are created in the epidermis to allow delivery of drugs to the blood vessels present in the dermal layer of the skin.
o Researchers report on a technique for administering contraceptive hormones through special backings on jewelry such as earrings, wristwatches, rings, or necklaces. The contraceptive hormones are contained in patches applied to portions of the jewelry in contact with the skin, allowing the drugs to be absorbed into the body (Georgia Institute of Technology, 2019).

### Possibilities for new means of drug development

⁃ Developing newer biotechnology-based cellular or molecular models that could better replicate reproductive processes.
⁃ Methods that act after ovulation and interfere with sperm delivery or function in the male or in the female genital tract or both ought to be adopted.
⁃ Design of nonhormonal contraceptive agents—as an alternative option to hormonal formulations—with the help of herbals.
⁃ New delivery mechanisms that can act both short and long term; the possibilities are to develop herbal pessary, jelly, patches, and condoms, or mechanical devices with natural ingredients to optimize the effects.
⁃ Methods which limit the side effects associated with systemic exposure should be developed in lower dosage forms to ensure efficacy.
⁃ Technologies that markedly improve the cost, acceptability, and deliverability of contraceptives.
⁃ Personalized contraception-human genome could minimize the side effects while maximizing health benefits at the individual level.

## Conclusion

Fertility and contraception are continued subjects of biomedical research and innovation. Alternatives to unmet needs for safer contraception methods and drugs are searched for. Many Ayurvedic medicinal ingredients and compound formulations are claimed to inhibit male and female fertility as mentioned in the classical literature. Several of these validated drugs possess spermicidal, antispermatogenic, antiovulatory, anti-implantation, antiestrogenic, and abortifacient activity. The Indian system of medicine, Ayurveda, offers highly promising opportunities when analytical, biological, technological, and clinical advances are collectively integrated with therapeutic rationale based on Ayurvedic principles. A plethora of available data, information, and knowledge on these ingredients could be the subject of newer research interests.

These medicinal ingredients need further reexamination and critical evaluation to explore their lesser known or unknown pharmacological and biological activity/activities and effects. Present-day biotechnological methods could be usefully utilized to evaluate their contraceptive efficacies. There is a need to revive and stimulate new research programs and projects that will not only benefit the need of contraception but will also throw new light on reproductive biology.
